# Monocytic-Myeloid Derived Suppressor Cells Suppress T-Cell Responses in Recovered SARS CoV2-Infected Individuals

**DOI:** 10.3389/fimmu.2022.894543

**Published:** 2022-06-24

**Authors:** Nadejda Beliakova-Bethell, Kathirvel Maruthai, Ruijie Xu, Liliana C. M. Salvador, Ankita Garg

**Affiliations:** ^1^ Department of Medicine, University of California San Diego, San Diego, CA, United States; ^2^ Veterans Administration (VA) San Diego Healthcare System and Veterans Medical Research Foundation, San Diego, CA, United States; ^3^ Department of Infectious Diseases, College of Veterinary Medicine, University of Georgia, Athens, GA, United States; ^4^ Institute of Bioinformatics, University of Georgia, Athens, GA, United States; ^5^ Center for the Ecology of Infectious Diseases, University of Georgia, Athens, GA, United States

**Keywords:** monocytic-myeloid derived suppressor cells, SARS CoV2 infection, T-cell responses, transcriptomic (RNA-Seq), cytokines

## Abstract

Coronavirus disease 2019 (COVID-19) caused by SARS Coronavirus 2 (CoV2) is associated with massive immune activation and hyperinflammatory response. Acute and severe CoV2 infection is characterized by the expansion of myeloid derived suppressor cells (MDSC) because of cytokine storm, these MDSC suppress T cell functions. However, the presence of MDSC and its effect on CoV2 antigen specific T cell responses in individuals long after first detection of CoV2 and recovery from infection has not been studied. We and others have previously shown that CD11b^+^CD33^+^CD14^+^HLA-DR^-/lo^ monocytic MDSC (M-MDSC) are present in individuals with clinical recovery from viral infection. In this study, we compared the frequency, functional and transcriptional signatures of M-MDSC isolated from CoV2 infected individuals after 5-months of the first detection of the virus (CoV2+) and who were not infected with CoV2 (CoV2-). Compared to CoV2- individuals, M-MDSC were present in CoV2+ individuals at a higher frequency, the level of M-MDSC correlated with the quantity of IL-6 in the plasma. Compared to CoV2-, increased frequency of PD1^+^, CD57^+^ and CX3CR1^+^ T effector memory (T_EM_) cell subsets was also present in CoV2+ individuals, but these did not correlate with M-MDSC levels. Furthermore, depleting M-MDSC from peripheral blood mononuclear cells (PBMC) increased T cell cytokine production when cultured with the peptide pools of immune dominant spike glycoprotein (S), membrane (M), and nucleocapsid (N) antigens of CoV2. M-MDSC suppressed CoV2 S- antigen-specific T cell in ROS, Arginase, and TGFβ dependent manner. Our gene expression, RNA-seq and pathway analysis studies further confirm that M-MDSC isolated from CoV2+ individuals are enriched in pathways that regulate both innate and adaptive immune responses, but the genes regulating these functions (*HLA-DQA1*, *HLA-DQB1*, *HLA-B*, *NLRP3*, *IL1β*, *CXCL2*, *CXCL1*) remained downregulated in M-MDSC isolated from CoV2+ individuals. These results demonstrate that M-MDSC suppresses recall responses to CoV2 antigens long after recovery from infection. Our findings suggest M-MDSC as novel regulators of CoV2 specific T cell responses, and should be considered as target to augment responses to vaccine.

**Graphical Abstract:**
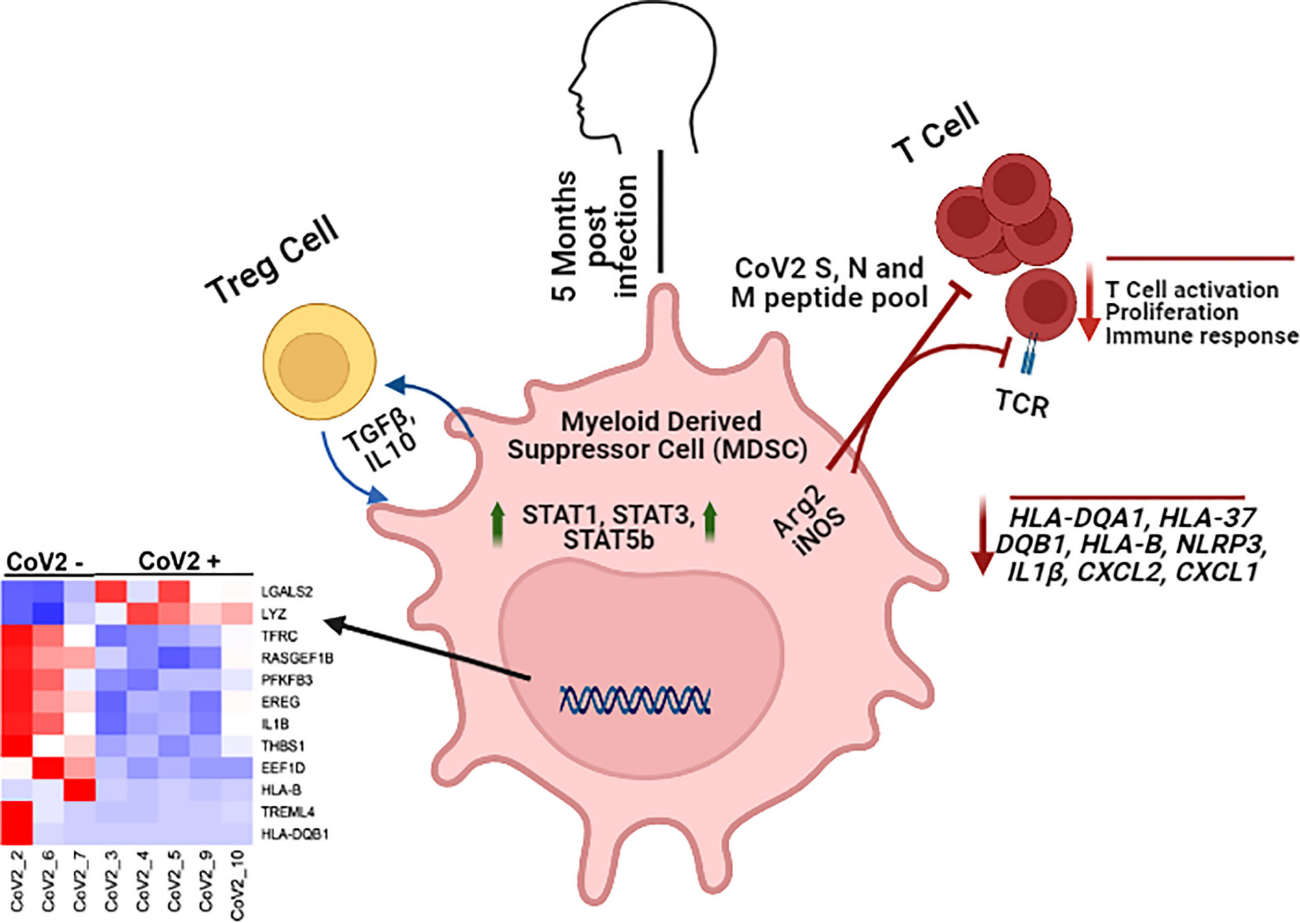
Monocytic MDSC inhibit CoV2 antigen specific T cell responses and maintain a divergent transcriptomic profile long after recovery from infection.

## Introduction

The coronavirus disease 2019 (COVID-19) pandemic caused by SARS CoV2 (CoV2) virus remains a global threat and cause of massive worldwide morbidity and mortality (https://www.who.int/emergencies/diseases/novel-coronavirus-2019). Even though the pathogenesis of COVID-19 remains elusive, ranging from asymptomatic to multiorgan failure and death, CoV2 infection is unarguably associated with massive immune activation and hyperinflammatory response because of the cytokine storm (1–6). A decline in T cell numbers (lymphopenia) in severe COVID-19 disease suggests the important role of these cells during CoV2 infection (3, 7–11). Furthermore, an association of early detection of CoV2 specific T cells with mild disease and late induction with severe disease support that T cells are indispensable for viral clearance (12–15). Experimental evidence shows that the induction of T cell responses to multiple immune dominant CoV2 antigens is a critical parameter of antiviral efficacy (12, 16–20). Although the kinetics and duration of cellular immunity in CoV2 infection are highly heterogeneous, CoV2 specific T cells persist for at least 6-8 months after natural infection (17, 21–27). The regulation of T cell responses/effector function during the contraction phase of T cells remains less explored.

Myeloid derived suppressor cells (MDSC) are a heterogeneous population of cells derived from immature myeloid progenitors and have immunosuppression potential (28–34). Although much extensively studied in cancer, increasing evidence demonstrate their importance in microbial infections including viral respiratory infections (29, 32, 35–37). In humans, MDSC expresses ommon myeloid markers (CD11b^+^CD33^+^HLA DR^-/lo^) and, depending on the presence of CD15 or CD14, are divided into granulocytic or monocytic subsets, respectively (28, 38–40). These cells utilize multiple mechanisms such as 1) depleting the microenvironment of important nutrients for T cell activation by expressing high levels of arginase-1 (Arg-1) and indoleamine 2,3 dioxygenase (IDO), 2) producing oxygen species such as nitric oxide (NO), reactive oxygen species (ROS) and peroxynitrite (PNT), 3) producing immunosuppressive cytokines such as TGF-β and IL-10, and 4) inducing expansion of regulatory T-cell (Treg) to suppress adaptive response (41–45). Expansion and function of MDSC is regulated by STAT family of transcription factors primarily STAT3 and STAT5, which induces the expression of anti-apoptotic genes and prevents differentiation of myeloid progenitor cells into mature myeloid cells (28, 42, 46–48). Considerable research, predominantly performed in animal models, has demonstrated inhibition of antitumor and antimicrobial activity by MDSC. Multiple recent studies have also shown activation and expansion of MDSC in severe CoV2 infection result in decreased numbers and impaired function of natural killer and T cells (35, 49–53). However, information regarding the presence of MDSC, and their effect on T cell function in individuals with past CoV2 infection is limited.

In this study, we investigated CD11b^+^CD33^+^CD14^+^HLA-DR^-/lo^ monocytic myeloid derived suppressor cells (M-MDSC) in individuals with a history of CoV2 infection (CoV2+) confirmed by RT PCR and individuals without CoV2 (CoV2-). We found an increased frequency of M-MDSC in the peripheral blood of CoV2+ as compared to CoV2-. Importantly, depleting M-MDSC augmented T cell cytokine production in response to the peptide pool of immune dominant CoV2 antigens. Our RNA sequencing (RNAseq) data reveals differential expression of genes in CoV2+ M-MDSC. These results provide insight that targeting M-MDSC may improve the cellular immune response to CoV2 vaccines.

## Materials and Methods

### Patient Population

Blood was obtained after receipt of written informed consent from BCG un-vaccinated, HIV-, CoV2– and CoV2+ persons. CoV2- and CoV2+ participants with clinical signs of CoV2 infection (temperature of at least 100.4°F, cough, shortness of breath, chills, sore throat, muscle pain, or new loss of taste or smell) or in close contact with anyone showing these symptoms at the time of sample collection were not enrolled for this study. All studies were conducted in accordance with the Declaration of Helsinki guidelines and approved by the institutional review board of the University of Georgia, Athens.

### Cell Isolation and Culture

Peripheral blood mononuclear cells (PBMC) were isolated from freshly obtained blood by Ficoll density centrifugation (GE Healthcare) and cultured in RPMI1640 supplemented with AB human serum. For some experiments, PBMC and MDSC depleted PBMC were cultured with the PepTivator® peptide pools for surface glycoprotein (S), nucleocapsid phosphoprotein (N), or membrane glycoprotein (M) of SARS-CoV2 as per the manufacturer’s instructions (Miltenyi Biotec). Briefly, 1.5 x10^6^ cells were cultured in a flat-bottom 96-well plate in a volume of 150 µl culture media, and peptide pools were added at a final concentration of 0.6 nmol of each peptide. To determine the effect of PD1, PD-L1, galectin, ROS, arginase, and iNOS cells were cultured with the peptide pool of S-protein in the presence or absence of neutralizing (PD1, PD-L1, and galectin) or isotype control antibodies, or chemical inhibitors (ROS, arginase, and iNOS).

### Antibodies and Other Reagents

Antibodies used for flow cytometry were Alexa Fluor488-anti-CD11b, PE-Dazzel594-anti-HLA DR, PE/Cy7-anti-CD14, APC-anti-CD33, PerCP-Cy5.5-anti-CD66b, BV421-anti-CD19, BV510/PE/Cy7-anti-CD3, APC-eF780/APC-anti-CD4, PE-Fire700/APC-eF780-anti-CD8, AF488-anti-CD45RA, BV421/BV605-anti-CD62L, AF700-anti-CD27, BV421-anti-CD28, PerCP-Cy5.5-anti-PD1, PE-Dazzel594-anti-CD57, PE-anti-CX3CR1, PE-anti-IL-2; LIVE/DEAD fixable aqua stain dye. For neutralization studies, monoclonal antibodies to PD-1, PD-L1, galectin or isotype (10 µg/ml) control were used (all from Biolegend). Chemical inhibitors used were ROS inhibitor catalase (100 U/mL), arginase inhibitor NG-monomethyl-L-arginineacetate (0.5 mM) (both from Sigma Aldrich), and iNOS inhibitor N( ϖ)-hydroxy-nor-L-arginine (0.5 mM; EMD Millipore Corp.).

### Depletion of MDSC From Fresh PBMC

Freshly isolated PBMC were stained for CD14, HLA DR, and LIVE/Dead Aqua stain; Aqua^+^ dead cells were excluded and CD14^+^HLA DR^-/lo^ MDSC depleted using flow cytometry were collected separately. MDSC depleted PBMC were cultured for flow cytometry and measurement of cytokines. Total RNA was isolated from MDSC for transcriptomic and quantitative reverse-transcription polymerase chain reaction (qRT-PCR) analysis.

### Immunolabeling and Flow Cytometry

Cells were stained for surface markers using respective antibodies and cell staining buffer. For intracellular IL-2, surface-stained cells were fixed and permeablized using a Fixation/Permeabilization kit and anti-IL2 antibody. Flow cytometry was done on Quanteon and data was analyzed using Flowjo. A minimum of 300,000 Aqua^-^ LIVE cells were collected for each sample. Controls for each experiment included unstained cells and fluorescence minus one (FMO).

### Quantification of Cytokines

Supernatants collected from PBMC and MDSC depleted PBMC cultures were stored at -80°C. Quantity of IFNγ, TNFα, IL-10, IL-17, IL-12p70 were determined at 24-hours post-stimulation with Peptivator Peptide pools using Bioplex analyte detection kit (Biorad). Plasma levels of IL-6 and IL-8 were determined using IL-6 Quantikine HS (sensitivity 0.09 pg/ml) and IL-8 Quantikine HS (sensitivity 0.4 pg/ml), respectively (both from R&D Systems)

### Quantitative Reverse-Transcription Polymerase Chain Reaction (qRT-PCR)

qRT-PCR was done as previously described (54). Total RNA was isolated from sorted CD14^+^HLA DR^-/lo^ MDSC from CoV- and CoV2+ individuals using TRIzol™ reagent (Thermo Fisher Scientific) according to the manufacturer’s protocol; 250 ng RNA was used for cDNA synthesis using iScript™ cDNA Synthesis Kit (BioRad, USA) according to the manufacturer’s instructions. qRT-PCR was performed using the PowerUp™ SYBR™ Green Master Mix (Thermo Fisher Scientific, USA) and data were acquired with the Applied Biosystems™ StepOne™ Real-Time PCR System (Applied Biosystems/Life Technologies, Germany). The Human acidic ribosomal protein (HuPO) was used as a housekeeping gene. The list of genes and primers used in this study is in **Supplementary Table 1**. Data were analyzed to calculate the relative quantification of the genes in comparison to the HuPO gene by comparative Ct method (2-ΔCt) (55).

### Statistical Analysis

Data are expressed as mean values ± standard error mean (SEM). Paired Student t-tests were used to determine the statistical significance of *in vitro* experiments. Comparisons between CoV2- and CoV2+ subjects was made by non-parametric Mann Whitney U test; comparisons between different parameters were analyzed using Spearman correlation. Statistical analysis was performed using Graphpad Prism 9 (La Jolla, CA); p-values of <0.05 were considered statistically significant.

### RNA-Seq Data Generation

CD14^+^HLA DR^-/lo^ cells from freshly isolated PBMC were isolated after excluding dead cells by flow cytometry. RNA was isolated using RNeasy Mini Kit (QIAGEN, Germany) and quality was assessed using Bioanalyzer (Agilent Technologies, USA). All samples were checked for RNA integrity number (RIN). The mean RIN for 10 samples was 6.9 with a standard deviation 2.9. RNA-Seq libraries were prepared in two batches using 10 pg -1 ng total RNA and SMART-Seq HT PLUS kit kit (Takara Bio USA, Inc.). All libraries were sequenced to the depth of average of 34294989 (range from 16694996 to 46416998) using Illumina 500 mid-output PE75 sequencer at the Georgia Genomics andBioinformatics Core (GGBC), University of Georgia, Athens.

### Differential Gene Expression and Pathway Analysis

Data were available from the GGBC, University of Georgia, Athens Core in the.fastq format. Data pre-processing included concatenating reads for one sample obtained on different lanes of the sequencer into one file. Filtering low quality reads and removal of the 3’ adapter sequences were further performed using the Trim Galore tool, which utilizes the Catadapt program (56). Reads were mapped to the latest version of the human genome hg38 (GRCh38.p13) using HISAT2 (57). Mapped reads were counted against the human GENCODE annotation (v37) (58) using HT-Seq (59). The *EdgeR* library (60) in the R computing environment was used for quality control of the RNA-Seq data, and *ComBat-seq* method (61) for correction of batch effects. Differential gene expression analysis was conducted using *EdgeR*. *EdgeR* uses empirical Bayes estimation and exact tests based on the negative binomial distribution of the RNA-Seq data, followed by false discovery rate (FDR) correction using the Benjamini-Hochberg method (62). Genes were considered differentially expressed when FDR-corrected *p*-values were <0.05, or nominal p-value <0.05 in an exploratory approach. Pathways over-represented for DEGs were identified using the Database for Annotation, Visualization and Integrated Discovery (DAVID) v6.8 (63, 64) and KEGG (65, 66) and Reactome (67) databases. For pathway analysis, all genes with a nominal p-value <0.05 were used as input. DAVID uses a one-tailed Fisher’s Exact test for gene set enrichment analysis. Pathways with nominal *p*-values <0.1 were considered significantly enriched for DEGs.

## Results

### Patient Characteristics

All the participants were unvaccinated for COVID-19. CoV2- participants self-reported their COVID-19 status; CoV2+ participants who self-reported positive RT PCR in past were included in the study. Participants were enrolled at a median of 163 days (5.4 months) from the first detection of CoV2 infection (**Table 1**). Our participant enrollment form included self-report for Type 2 diabetes (T2D), hypertension, tuberculosis, smoking and chewing tobacco, any respiratory complications, or any other ongoing medical condition. Of all the CoV2- and CoV2+ participants, 2 CoV2- individuals and 1 CoV2+ reported T2D and/or hypertension, and 1 CoV2+ participant self-reported suffering from anxiety. None of the participants reported any other co-morbidity.

**Table 1:** Demographic and clinical variables in study particpants.

	CoV2- (N = 9)	CoV2+ (N = 13)	p-value
**Age in years** **Median (Range)**	49 (24-61)	33 (22-51)	0.59
**Gender distribution (Number)** ** Female** ** Male**	4 5	6 8	
**Time to CoV2+ (Days)** **Median (Range)**	-	163 (18-313)	-

### Increased Frequencies of M-MDSC in CoV2+ Individuals

**Figure 1:**
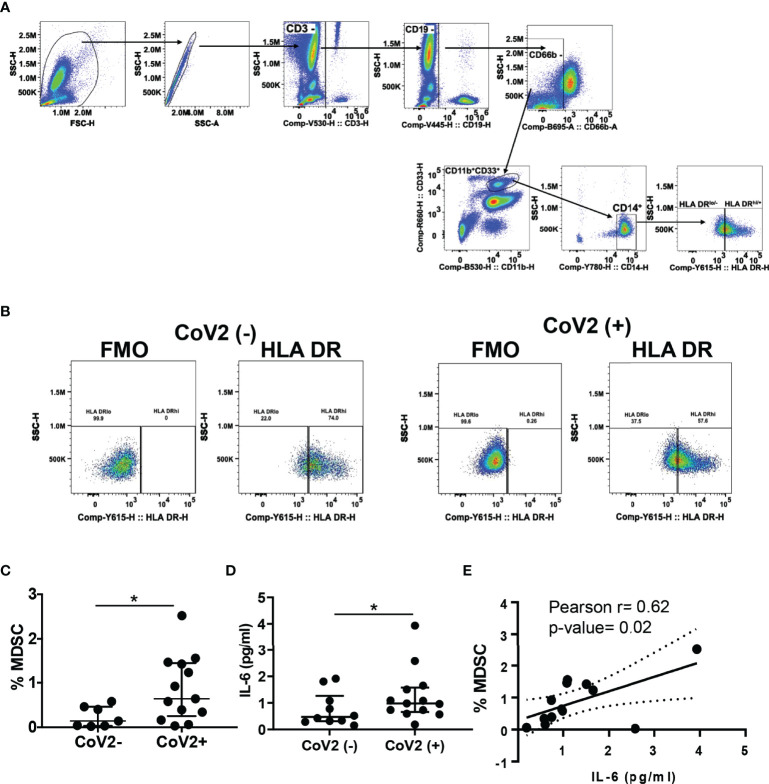
M-MDSC expansion in CoV2+ individuals is dependent on IL-6: **(A–C)** Heparinized blood obtained from CoV2- and CoV2+ individuals was stained with anti-CD3, -CD19, -CD11b, -CD33, -CD14, CD66b, -HLA DR antibodies, cells were analyzed as CD3^-^CD19^-^CD66b^-^CD11b^+^CD33^+^CD14^+^HLA DR^-/lo^ by flow cytometry. **(A)** Gating strategy for MDSC is shown **(B)** A representative dot plot with HLA DR-/lo region from CoV2(-)and CoV2(+)is shown. **(C)** Percentages of MDSC are shown. **(D)** The quantity of cytokine IL-6 in the plasma of CoV2- and CoV2+ individuals was measured by ELISA, as in Methods. **(E)** Plasma IL-6 quantity was correlated with the circulating frequency of M-MDSC in CoV2+ individuals. **(C, D)** Each dot in the plots depicts data of each individual donor, the plots include observations from 25^th^ to 75^th^ percentile. The horizontal line represents the median value. **(D)** Each dot in the plot depicts data of each individual donor; black solid and dotted lines, model-estimated values, and their 95% confidence intervals. *p < 0.05.

Severe COVID-19 is associated with increased circulating MDSC, which declines upon clinical recovery from the disease (35, 49, 50, 52). Here we sought to investigate the presence of M-MDSC at a median of 163 days (approx. 5 months) from the first detection of CoV2 infection by RT PCR. We found an increased frequency of M-MDSC in peripheral blood of CoV2+ individuals as compared to CoV2- controls (0.2±0.09 vs 0.9±0.2; p=0.03) (**Figures 1A–C**). Of note, the M-MDSC frequency in peripheral blood of one CoV2- participant who self-reported as hypertensive was relatively higher (0.4%) as compared to other CoV2- participants. We have previously shown that M- MDSC expansion during viral infection is dependent on IL-6 (32, 68). Additionally, COVID-19 is associated with elevated levels of IL-6. Therefore, we quantified IL-6 in the plasma of CoV2- and CoV2+ individuals. Despite a long time after recovery from CoV2 infection, we found higher levels of IL-6 in plasma of CoV2+ individuals, compared to CoV2- controls (0.76±0.2 vs 1.3±0.3; p=0.04) (**Figure 1D**). Furthermore, a positive correlation was found between the quantity of plasma IL-6 and the circulating frequency of MDSC (Pearson r= 0.62; p=0.02) (**Figure 1E**). Of note, IL-8 was also elevated in CoV2+ individuals, but its level did not correlate with M-MDSC (**Supplementary Figure 1A**). The plasma IL-6 quantity of the hypertensive CoV2- participant was not higher than the remaining participants.

### Phenotypic Evidence of T Cell Exhaustion and Senescence in CoV2+ Individuals

**Figure 2:**
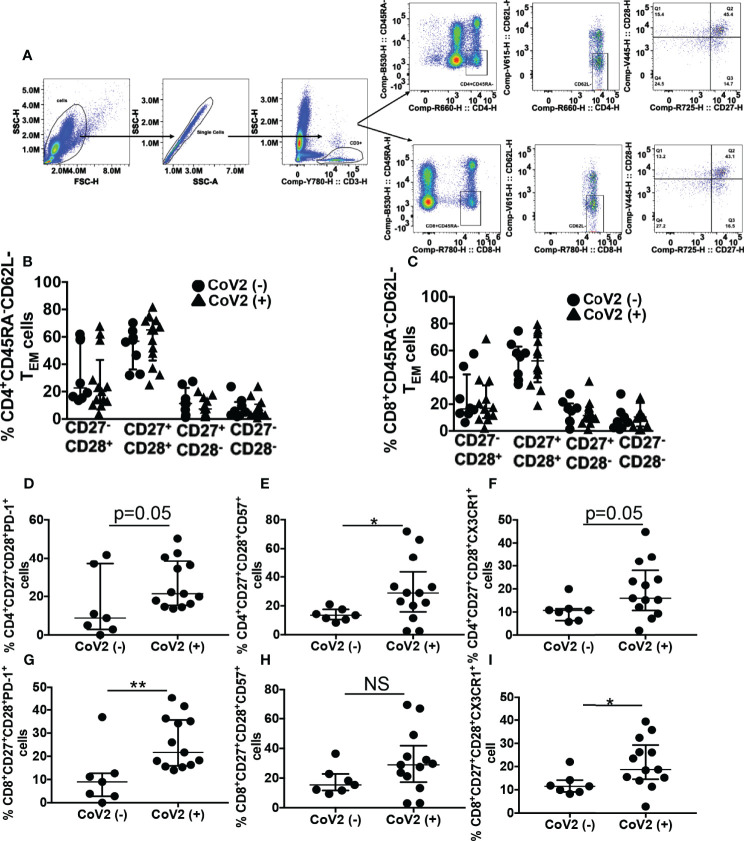
CoV2+ individuals exhibit an aberrant T cell phenotype 5 months after the first detection of infection: Heparinized blood obtained from CoV2- and CoV2+ individuals was stained with anti-CD3, -CD4, -CD8, -CD45RA, -CD62L, CD27, -CD28, -CD57, -PD-1 and -CX3CR1 antibodies. **(A–C)** Cells were analyzed for various T memory cell subsets as indicated. **(A)** Gating strategy for T cells is shown **(B, C)** Percentages of T memory cells are shown. **(D–F)** Percentages of PD-1^+^, CD57^+^ and CX3CR1^+^ cells in CD3^+^CD4^+^CD45RA^-^CD62L^-^CD27^+^CD28^+^ T memory cell subset **(G–I)** Percentages of PD-1^+^, CD57^+^ and CX3CR1^+^ cells in CD3^+^CD8^+^CD45RA^-^CD62L^-^CD27^+^CD28^+^ T memory cell subset is shown. For all, each dot in the plots depicts data of each individual donor, the plots include observations from 25^th^ to 75^th^ percentile. The horizontal line represents the median value. *p < 0.05; **p < 0.005 NS, Non-significant.

Effector memory T (T_EM_) cells remain long-term after an infection is eliminated. These T cell subsets are critical to eradicating virus by their ability to produce anti-viral cytokines to control viral replication. Apart from the expression of lymph node homing receptors CCR7 and CD62L, CD27 and CD28 along with CD45RA can be used to discriminate naïve and T_EM_ cells with CD45RA^+^CD27^+^CD28^+^ (naïve T cells), CD45RA^-^CD62L^-^CD27^+^CD28^+^ (T_EM_) (69–72). Memory T cells with senescent and exhaustion phenotype have been reported during severe CoV2 infection, displaying a reduced capacity for antiviral cytokine production (73–75). In this research, we sought to determine if phenotypic T cell abnormalities persist 5 months from the first detection of CoV2 infection. For this, initially, we compared the frequency of various CD45RA^-^CD62L^-^ T_EM_ subsets present in the CD4^+^ and CD8^+^ compartments of the whole blood of CoV2- and CoV2+ individuals. We found a comparable frequency of CD27^-^CD28^+^, CD27^+^CD28^+^, CD27^+^CD28^-^, and CD27^-^CD28^-^ T_EM_ cell subset in the CD4^+^ and CD8^+^ compartments of the two groups (**Figures 2A–C**, and **Tables 2**, **3**). The hypertensive CoV2- participant with a higher frequency of MDSC also exhibited elevated frequencies of CD4^+^ and CD8^+^ CD27^-^CD28^+^ cells.

**Table 2:** Percentage of CD4 T effector memory (T_EM_) cell subsets in the peripheral blood of study participants.

	CoV2- (N = 9)	CoV2+ (N = 13)	p-value
**CD27^-^CD28^+^**	33.6 ± 7.7	26.8 ± 5.9	0.2
**CD27^+^CD28^-^**	12.6 ± 3.4	8.3 ± 1.8	0.5
**CD27^-^CD28^-^**	8.0 ± 2.6	7.2 ± 1.8	0.9
**CD27^+^CD28^+^** **PD1^+^** **CD57^+^** **CX3CR1^+^**	52.3 ± 4.9	57.7 ± 4.9	0.4
	15.2 ± 6.4	26.5 ± 3.5	*0.05*
	13.8 ± 1.6	30.6 ± 6	*0.036*
	10.7 ± 1.7	19.7 ± 3.3	*0.05*

Statistical significant values are italicized.

**Table 3:** Percentage of CD8 T effector memory (T_EM_) cell subsets in the peripheral blood of study participants.

	CoV2- (N = 9)	CoV2+ (N = 13)	p-value
**CD27^-^CD28^+^**	24.4 ± 6.5	22.2 ± 5.3	0.7
**CD27^+^CD28^-^**	14.2 ± 3	13.7 ± 2.7	0.9
**CD27^-^CD28^-^**	8.9 ± 3	11.5 ± 2.5	0.7
**CD27^+^CD28^+^** **PD1^+^** **CD57^+^** **CX3CR1^+^**	53.2 ± 4.8	52.6 ± 5.5	0.9
	10.9 ± 4.6	25.9 ± 3	*0.005*
	18 ± 3.5	30.9 ± 5.7	0.1
	12.4 ± 1.8	21.6 ± 2.9	*0.02*

Statistical significant values are italicized.

Of the four T_EM_ subsets identified, the frequency of CD27^+^CD28^+^ was highest in CD4^+^ and CD8^+^ T cells. Therefore subsequently, we compared PD1^+^, CD57^+^, and CX3CR1^+^ cells in these T_EM_ subsets of CD4^+^ and CD8^+^ compartments. As compared to CoV2- individuals, CoV2+ individuals exhibited increased frequency of CD4^+^CD57^+^ T_EM_ cells (13.8±1.6 vs 30.6±6; p=0.04). Even though the frequency of CD4^+^PD1^+^ and CD4^+^CX3CR1^+^ T_EM_ cells was also higher in CoV2+ individuals, it was less stringent than CD57^+^ cell frequency (**Figures 2D–F** and **Table 2**). In contrast to CD4^+^ T_EM_ cells, CoV2+ individuals exhibited increased frequency of CD8^+^PD1^+^ and CD8^+^CX3CR1^+^ cells (10.9±4.6 vs 25.9±3; p=0.005 and 12.4±1.8 vs 21.6±2.9; p=0.02, respectively) (**Figures 2G–I** and **Table 3**). Of note, the frequency of CD4^+^CD57^+^ T_EM_ cells, and CD8^+^PD1^+^ or CD8^+^CX3CR1^+^ T_EM_ cells did not correlate with the circulating MDSC (Data not shown). Collectively these studies suggest persistence of T cells linked to the suppression of their response for long-duration post CoV2 infection.

### M-MDSC Regulates CoV2 Specific T Cell Cytokine Production

**Figure 3:**
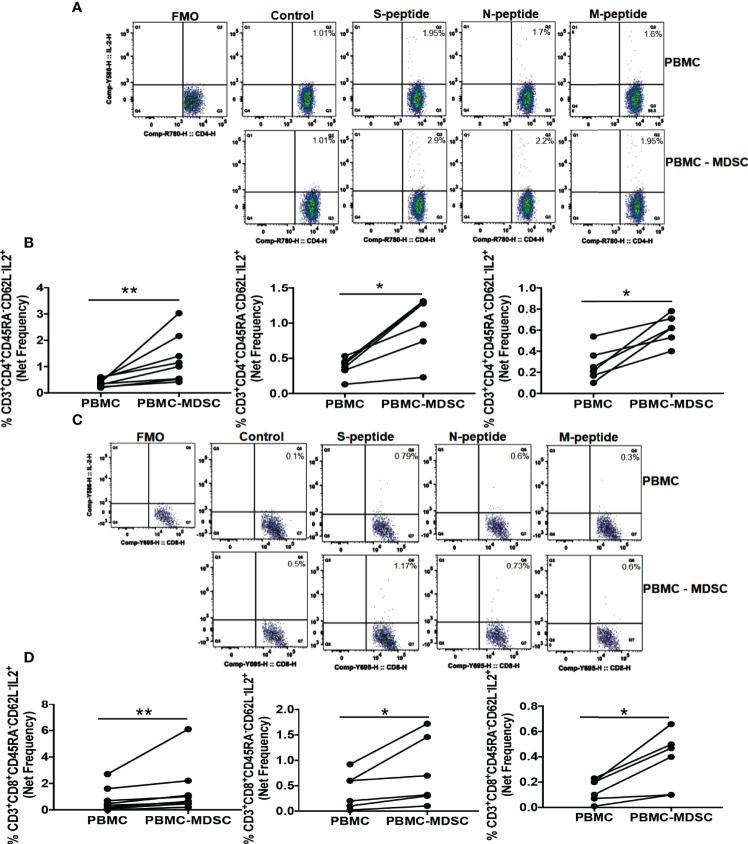
M-MDSC regulates CoV2 antigen-specific IL-2 production: Freshly isolated PBMC from CoV2+ individuals were stained with anti-CD14 and -HLA DR antibodies; CD14^+^HLA DR^-/lo^ M-MDSC were depleted from PBMC by flow cytometry. Whole PBMC (PBMC) and MDSC depleted PBMC (PBMC-MDSC) were cultured in the absence or presence of peptide pools of S-, N-, and M- antigens of CoV2 for 20-24 hours. Cells were stained with anti-CD3, -CD4, -CD8, -CD45RA, -CD62L, IL-2 antibodies, and LIVE/DEAD fixable stain. **(A, B)** Percentages of CD3^+^CD4^+^CD45RA^-^CD62L^-^IL-2^+^ cells was determined. **(C, D)** Percentages of CD3^+^CD8^+^CD45RA^-^CD62L^-^IL-2^+^ cells was determined. **(A, C)** Representative dot plots showing CD4^+^IL-2^+^ gated on Live CD3^+^CD4^+^CD45RA^-^CD62L^-^ **(A)**, and CD8^+^IL-2^+^ gated on Live CD3^+^CD8^+^CD45RA^-^CD62L^-^ **(C)** are shown. **(B, D)** Each dot represents an individual donor. *p < 0.05, **p < 0.005, FMO, Fluorescence minus one.

We found increased MDSC five months post-CoV2 infection (**Figure 1B**). In the next set of experiments, we sought to investigate if these MDSC regulate T cell function in response to CoV2 antigens. For this, we depleted CD14^+^HLA DR^lo/-^ MDSC from freshly isolated PBMC of CoV2- and CoV2+ individuals, and stimulated whole PBMC and MDSC depleted PBMC with peptide pools of S, N, and M antigens for 16-18 hours. The frequency of CD3^+^CD45RA^-^CD62L^-^ CD4^+^ and CD8^+^ T_EM_ cells was determined by flow cytometry. We have previously established that depleting CD14^+^HLA DR^lo/-^ MDSC does not change CD3^+^ T cell percentage or expression of CD69, CD38, and HLA DR T cell activation markers in MDSC depleted PBMC fraction (76). In this research, we found that the net CoV2 specific frequency of CD3^+^CD45RA^-^CD62L^-^CD4^+^IL-2^+^ was greater in MDSC depleted PBMC cultures when compared to whole PBMC cultures of individuals with past CoV2 infection (p-values 0.007 for S peptide pool, 0.03 for N and M peptide pools) (**Figures 3A, B**). Similarly, the net CoV2 specific frequency of CD3^+^CD45RA^-^CD62L^-^CD8^+^IL-2^+^ was greater in MDSC depleted PBMC cultures when compared to whole PBMC cultures of individuals with past CoV2 infection (p-values 0.008 for S peptide pool, 0.03 for N and M peptide pools) (**Figures 3C, D**). The cultures of CoV2- individuals did not produce significant IL-2 in response to CoV2 antigens (data not shown).

**Figure 4:**
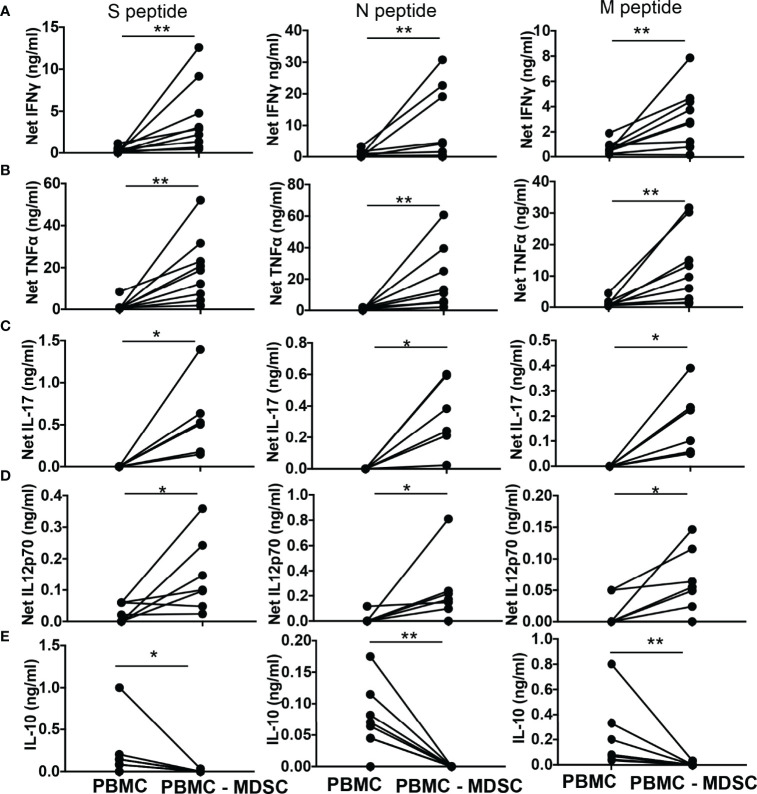
M-MDSC regulates CoV2 antigen-specific T cell cytokine production: **(A–E)** Freshly isolated PBMC from CoV2+ individuals were stained with anti-CD14 and -HLA DR antibodies; CD14^+^HLA DR^-/lo^ M-MDSC were depleted from PBMC by flow cytometry. Whole PBMC (PBMC) and MDSC depleted PBMC (PBMC-MDSC) were cultured in the absence or presence of peptide pools of S-, N-, and M- antigens of CoV2 for 48-72 hours. Culture supernatant was stored at -80°C until further use. The quantity of cytokines in the culture supernatant was measured using Bio-Plex multiplex immunoassay system. For all, each dot represents an individual donor. *p < 0.05, **p < 0.005.

Subsequently, we quantified T helper cell (T_H_) cytokines, cytokines that activate T cells, and immune suppressive cytokine IL-10 in the culture supernatants of whole PBMC and MDSC depleted PBMC stimulated for 48-72 hours with peptide pool of CoV2 antigen. The net IFNγ (p-values 0.004 for S and N peptide pools, 0.008 for M peptide pool), TNFα (p-values 0.004 for S, N, and M peptide pools), IL-17 (p-values 0.03 for S, N, and M peptide pools), and IL-12p70 (p-values 0.04 for S peptide pool, 0.03 N and M peptide pools) produced by MDSC depleted PBMC cultures was greater when compared to whole PBMC cultures (**Figures 4A–D**). In contrast, MDSC depleted PBMC cultures when stimulated with CoV2 antigen peptides produced less IL-10 when compared to whole PBMC cultures stimulated identically (p-values 0.03 for S peptide pool, 0.008 for N and M peptide pools) (**Figure 4E**). These data establish that CD14^+^HLA DR^lo/-^ MDSC regulates the immunologic response to CoV2 and suppresses T_H_1 responses even after 5-6 months from initial exposure to the virus. The cultures of CoV2- individuals did not produce significant IFNγ in response to CoV2 antigens (**Supplementary Figure 1C**).

### M-MDSC Mediated T Cell Inhibition Is Dependent on Arg and ROS

**Figure 5:**
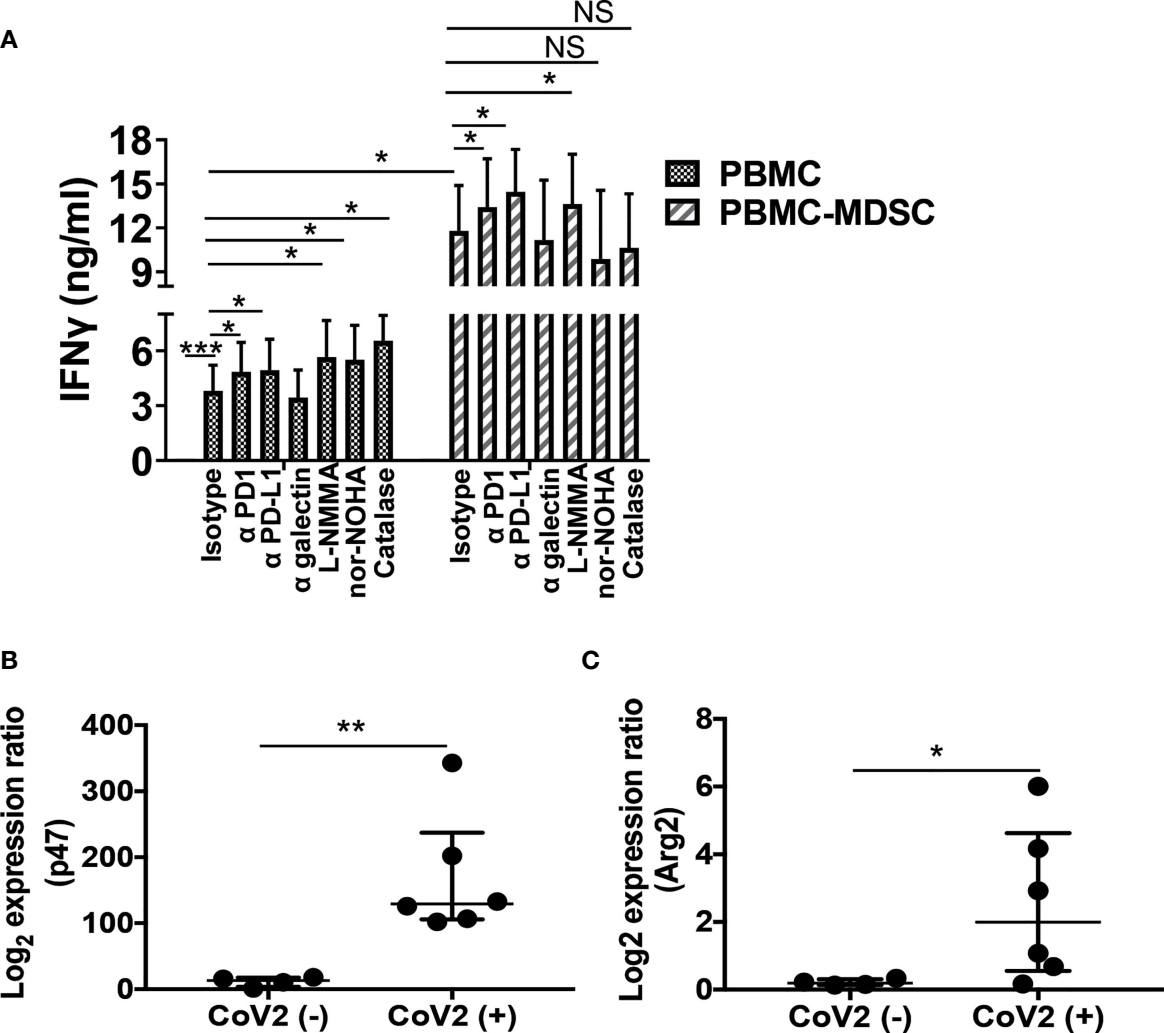
M-MDSC mediated T cell inhibition is dependent on Arg and ROS: Freshly isolated PBMC from CoV2- and CoV2+ individuals were stained with anti-CD14 and -HLA DR antibodies; CD14^+^HLA DR^-/lo^ M-MDSC were depleted from PBMC by flow cytometry. **(A)** Whole PBMC (PBMC) and MDSC depleted PBMC (PBMC-MDSC) of CoV2+ individuals were cultured in the absence or presence of the peptide pool of S-antigen in presence of blocking anti-PD1, -PD-L1, -galectin antibodies or isotype-matched control antibody, or in presence of iNOS inhibitor (L-NMMA), arginase inhibitor (nor-NOHA) or ROS inhibitor (Catalase). Supernatants were collected at 48-72 hours and the quantity of IFNγ was measured by ELISA. **(B, C)** RNA was isolated from sorted CD14^+^HLA DR^-/lo^ MDSC using Trizol. The expression of **(B)** *p47^phox^* (p47) and **(C)** *Arg2* was determined by qRT PCR as detailed in *Methods*. For **(A)** Mean values +/- SEM are shown from 4 separate donors; **(B, C)** each dot in the plots depicts data of each individual donor, the plots include observations from 25^th^ to 75^th^ percentile. The horizontal line represents the median value. *p < 0.05, **p < 0.005; ***p < 0.0005; NS, Non-significant.

MDSC utilize multiple mechanisms to confer their suppressor activity. These include biochemical and metabolic mediators ROS, iNOS, and arginase or cell surface inhibitory receptors PD-1, PD-L1, and α-galectin (28, 31, 35, 42, 43, 47). Here we sought to investigate if these mediators regulate T cell response, and delineate which of these mechanisms is utilized by MDSC to control T cell function in CoV2+ individuals. For this, initially, we depleted CD14^+^HLA DR^lo/-^ MDSC from freshly isolated PBMC of CoV2+ individuals, and cultured whole PBMC and MDSC depleted PBMC with peptide pool of S-antigen in the presence or absence of blocking antibodies for PD-1, PD-L1, or galectin-9 or iNOS, arginase, or ROS inhibitor. The quantity of IFNγ was quantified in the culture supernatants at 48-72 hours by ELISA. Compared to unstimulated controls, PBMC cultured with S-antigen produced more IFNγ (undetectable vs 3.8 ± 1.4 ng/ml; p=0.0001). This was further increased upon blocking PD-1 or PD-L1 (3.8 ± 1.4 vs 4.8 ± 1.5 ng/ml; p=0.01 and 3.8 ± 1.4 vs 4.9 ± 1.6 ng/ml; p=0.02, respectively), or inhibiting iNOS, arginase, or ROS (3.8 ± 1.4 vs 5.6 ± 2.1 ng/ml; p=0.05, 3.8 ± 1.4 vs 5.5 ± 1.9 ng/ml; p=0.05, and 3.8 ± 1.4 vs 6.5 ± 1.4 ng/ml; p=0.04). Of note, blocking galectin-9 did not affect IFNγ production (3.8 ± 1.4 vs 3.5 ± 1.5 ng/ml; p=0.59) (**Figure 5A**).

As previously observed (**Figure 4A**) compared to whole PBMC, IFNγ production was more in the culture supernatants of MDSC depleted PBMC cultures (3.8 ± 1.4 vs 11.8 ± 3 ng/ml; p=0.04). To explore the relative contribution of cell surface inhibitory receptors and biochemical mediators on the function of CoV2+ MDSC, blocking antibodies or biochemical inhibitors were added to MDSC depleted PBMC cultures, and the quantity of IFNγ was compared to MDSC depleted PBMC cultured without these inhibitors. We hypothesized that functionally active pathways in MDSC of CoV2+ individuals that may inhibit IFNγ production will be absent in MDSC depleted PBMC cultures. Therefore, the addition of antibodies or chemical inhibitors will have no effect on the IFNγ production in response to CoV2 antigen. However, similar to the whole PBMC culture, blocking PD-1, PD-L1, or inhibiting iNOS also increased IFNγ production in MDSC depleted PBMC cultures (11.8 ± 3 vs 13.4 ± 3.4 ng/ml; p=0.03, 11.8 ± 3 vs 14.5 ± 2.9 ng/ml; p=0.05, and 11.8 ± 3 vs 13.6 ± 3.4 ng/ml; p=0.03), suggesting that none of these pathways contribute to MDSC mediated T cell inhibition. Of note, inhibiting arginase or ROS did not affect IFNγ production (11.8 ± 3 vs 9.8 ± 4.7; p=0.41, and 11.8 ± 3 vs 10.6 ± 3.8; p=0.42, respectively) (**Figure 5A**), suggesting that M-MDSC are primarily responsible for arginase and ROS mediated IFNγ inhibition in PBMC cultures.

Concomitantly, we determined the gene expression of *PD-1*, *PD-L1*, *NOS2*, *Arg 1*, *Arg 2*, and *p47^ph^* ^ox^ subunit of ROS producing enzyme NADPH oxidase in MDSC isolated from CoV2+ and CoV2- individuals. Compared to CoV2- controls, MDSC isolated from CoV2+ individuals exhibited an increased expression of *p47^phox^* and *Arg 2* (11.4 ± 3.7 vs 168.7 ± 37.8; p=0.009, and 0.2 ± 0.04 vs 2.5 ± 0.9; p=0.03, respectively) (**Figures 5B, C**). Of note, the expression of *PD-1*, *PD-L1*, *NOS2*, and *Arg 1* remained undetectable. These results corroborated with our ELISA results. Specifically, neutralizing PD-1, PD-L1, and iNOS increased, whereas the addition of arginase or ROS inhibitors to MDSC depleted PBMC cultures do not affect IFNγ production. Collectively, these findings suggest that: (1) T cells utilize multiple mechanisms to control T_H_1 responses, and (2) MDSC utilizes arginase and ROS pathways to inhibit T cell function in CoV2+ individuals.

### CoV2 Infection Induces Long-Term Changes in Gene Expression Profiles

To determine whether infection with CoV2 has a long-term effect on gene expression in M-MDSC, we conducted an RNA-Seq study using samples from ten study participants (5 CoV2- and 5 CoV2+), that were profiled in two separate batches. Our quality control procedures identified a strong batch effect in the data (**Supplemental Figure 2**). Following batch effect correction and normalization, two outlier samples, one in each batch were identified, both of which from the CoV2- group. None of these outliers reported any co-morbidities. These outliers were removed based on their relative log expression distributions, resulting in a dataset of eight samples with a similar distribution pattern (**Supplemental Figure 2**), which was used for the subsequent differential gene expression analysis. Results from the differential gene expression analysis are shown in **Supplementary Table 2**.

**Figure 6:**
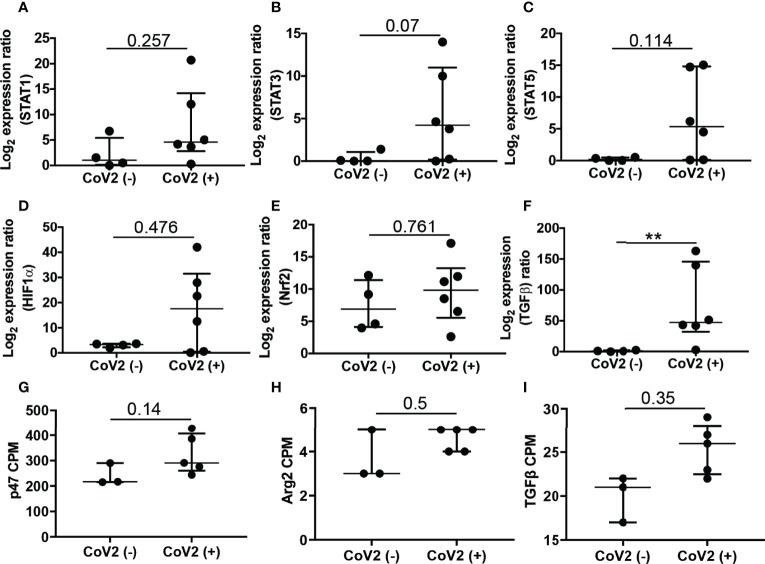
Expression of genes associated with expansion and function of M-MDSC: Freshly isolated PBMC from CoV2- and CoV2+ individuals were stained with anti-CD14 and -HLA DR antibodies; CD14^+^HLA DR^-/lo^ M-MDSC were sorted by flow cytometry and stored in Trizol. **(A–F)** RNA was isolated and the expression of indicated genes was determined by qRT PCR as detailed in *Methods*. **(G–I)** Expression of indicated genes was plotted from RNA-Seq data, as counts per million (cpm). For all, each dot in the plots depicts data of each individual donor, the plots include observations from 25^th^ to 75^th^ percentile. The horizontal line represents the median value. p-values are shown, **p < 0.005; nominal p-values are indicated for the RNA-Seq data.

First, we were interested in assessing the expression of genes quantified in a group of independent study participants by qRT PCR (**Figures 5**, **6**). Gene expression results of qRT PCR demonstrate significantly increased expression of *p47^phox^*, *Arg2* (**Figures 5B, C**), and *TGFβ* (**Figure 6F**) in M-MDSC isolated from CoV2+ as compared to CoV2- participants. Additionally, we considered expression levels of transcription factors *STAT1, STAT3, STAT5b, HIF-1α*, and *Nrf2* reported to regulate the expansion and function of MDSC. The median expression of all the genes was greater in M-MDSC isolated from CoV2+ participants, but the differences did not achieve statistical significance (**Figures 6A–E**). Of note, the expression of *STAT3* in M-MDSC of CoV2+ when compared to CoV2- participants approached significance (0.4 ± 0.3 vs 15.9 ± 10.6; p=0.07). Collectively, our findings of positive correlation between IL-6 and frequency of M-MDSC (**Figure 1C**) and *STAT3* expression (**Figure 6B**) suggest that the IL-6-STAT3 axis drives M-MDSC expansion in CoV2 infection. Contrary to the results obtained using qRT PCR, differences in the expression of genes between CoV2- and CoV2+ samples did not achieve statistical significance in the RNA-seq study. Nevertheless, some trends in expression remained the same, for example, *p47^phox^*, *Arg2* and *TGFβ* were upregulated when quantified by both qRT PCR and RNA-seq (**Figures 6G–I**), and *PD-1*, *PD-L1*, *NOS2* and *Arg1* were undetectable by either method.

**Figure 7:**
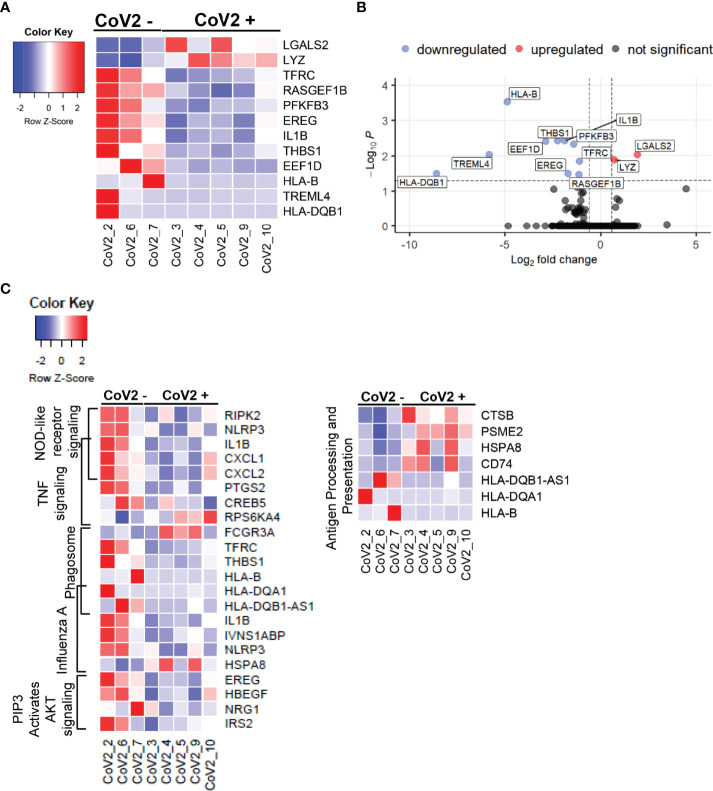
CoV2 infection induces long-term changes in global gene expression profiles: Differentially expressed genes in M-MDSC isolated from CoV2- and CoV2+ individuals were identified using *EdgeR*. **(A)** Heatmap was constructed using function *heatmap.2* from the library *gplots* in R. For the heatmap construction, samples were arranged by group (Cov2- followed by CoV2+), and genes were arranged by the average fold change in expression CoV2+/CoV2- (greatest to least); construction of dendrograms was omitted. Gene expression was scaled by row; scale bar represents scaled gene expression values. **(B)** Volcano plot was constructed using *EnhancedVolcano* function from the library *EnhancedVolcano* in R. The input for the volcano plot consisted of gene symbols along with logFC and FDR from the *EdgeR* differential gene expression analysis output. Vertical dash lines represent the fold change cut off (greater than 1.5 or less than -1.5); horizontal dash line represents the FDR cut off (less than 0.05). **(C)** Heatmaps were constructed for genes (nominal p < 0.05) from selected pathways (indicated on the left of the heatmaps). Samples were arranged by group (Cov2- followed by CoV2+). Genes were arranged by pathway; genes mapped to two pathways were added to the heatmap once at their overlap.

We next took the advantage of the RNA-seq data set to explore additional long-term effects of CoV2 infection on gene expression profiles. This analysis identified 12 new DEGs (FDR <0.05), of which 2 were upregulated and 10 were downregulated (**Figures 7A, B**); and a total of 188 DEGs with nominal p-value cut off of 0.05, of which 63 were up- and 125 downregulated (**Supplementary Table 2**). Remarkably, despite prolonged time after detected CoV2 (median 163 days/5 months), the absolute fold changes in the expression of most of these genes were quite high, 2- to 378-fold (**Supplementary Table 2**). Many of these genes were previously implicated in severe CoV2 infection; including some genes that did not reach statistical significance in our study. We show that: 1) some of these genes remained upregulated 5 months after infection (*LYZ*, *PLAC8* [fold increase 1.6; p=0.07)], 2) were upregulated during severe disease and downregulated 5 months after recovery (*PFKB3*, *CXCL8* (fold decrease 1.93; p=0.06), *TREML4*, *THBS1, EREG*), or 3) remained downregulated long-term (*IL1B*, *NLRP3*) (77–81). Our RNA-seq findings combined with cytokine studies (**Figures 3**, **4**) suggest that after recovery from COVID-19 disease, M-MDSC may contribute to control cytokine induced hyper inflammation, but retain their capacity to suppress T_H_1 cell function, critical for the anti-viral immune mechanism.

### Immune Response Pathways Are Enriched for Differentially Expressed Genes (DEGs) Modulated Long-Term After CoV2 Infection

We next explored the long-term effects of CoV2 infection on biological processes by conducting pathway enrichment analysis using KEGG and the Reactome databases. In total, this analysis identified 38 pathways significantly enriched for DEGs (nominal p-value < 0.1, **Supplementary Table 3**). Of these, ten pathways are directly related to the regulation of immune function, in particular regulating T cell responses (**Table 4**). Of note, the genes responsible for upregulated T cell function such as *HLA-B*, *HLA DQB1*, and innate immune signaling *EREG*, *IL1B*, *NLRP3* remained downregulated in M-MDSC of CoV2+ participants (**Figure 7C**). These findings suggest M-MDSC retains the capacity to regulate anti-CoV2 immune function long after CoV2 infection.

**Table 4:** Pathways enriched for DEGs identified between CoV2+ and CoV2- groups (nominal p-value < 0.05).

Category	Term	Genes	p-value
KEGG_PATHWAY	hsa04612:Antigen processing and presentation	*HSPA8*, *CD74*, *HLA-B*, *PSME2*, *HLA-DQA1*, *CTSB*, *HLA-DQB1*	0.0002
REACTOME_PATHWAY	R-HSA-114608:R-HSA-114608:Platelet degranulation	*TGFB1*, *HSPA5*, *SERPING1*, *TAGLN2*, *PPBP*, *THBS1*, *F5*	0.0019
KEGG_PATHWAY	hsa04621:NOD-like receptor signaling pathway	*RIPK2*, *IL1B*, *NLRP3*, *CXCL1*, *CXCL2*	0.0033
KEGG_PATHWAY	hsa04668:TNF signaling pathway	*RPS6KA4*, *IL1B*, *CXCL1*, *PTGS2*, *CXCL2*, *CREB5*	0.0064
KEGG_PATHWAY	hsa04145:Phagosome	*FCGR3A*, *TFRC*, *HLA-B*, *THBS1*, *HLA-DQA1*, *HLA-DQB1*	0.0247
KEGG_PATHWAY	hsa05164:Influenza A	*IVNS1ABP*, *HSPA8*, *IL1B*, *NLRP3*, *HLA-DQA1, HLA-DQB1*	0.0428
REACTOME_PATHWAY	R-HSA-1257604:R-HSA-1257604:PIP3 activates AKT signaling	*IRS2*, *NRG1*, *EREG*, *HBEGF*	0.0475
KEGG_PATHWAY	hsa05168:Herpes simplex infection	*CD74*, *EEF1D*, *IL1B*, *HLA-B*, *HLA-DQA1*, *HLA-DQB1*	0.0512
REACTOME_PATHWAY	R-HSA-877300:R-HSA-877300:Interferon gamma signaling	*HLA-B*, *TRIM21*, *HLA-DQA1*, *HLA-DQB1*	0.0582
REACTOME_PATHWAY	R-HSA-202424:R-HSA-202424:Downstream TCR signaling	*RIPK2*, *PSME2*, *HLA-DQA1*, *HLA-DQB1*	0.0807

Pathway enrichment analysis was conducted using the Database for Annotation, Visualization and Integrated Discovery (DAVID) v6.8 and KEGG and Reactome databases. Pathways with nominal p-value < 0.1 were considered significant. Pathways identified from both databases were combined and sorted by the nominal p-value.

## Discussion

Over the past couple of years, CoV2 infection has been a cause of mortality worldwide. With the development of various vaccines, hospitalization is reduced significantly in vaccinated individuals, yet the factors that may influence prolonged vaccine efficacy remain ill-defined (82–86). In this research, we demonstrate that elevated frequency of M-MDSC persists in peripheral blood of CoV2+ individuals even after 5-months post infection, as a result of persistently elevated levels of IL-6. We have also shown that these M-MDSC are functionally active, as depleting them increased the T cell cytokine production in response to CoV2 peptide pools. We show that M-MDSC suppresses T cell cytokine production through mechanisms involving ROS and arginase. Using the transcriptomic and pathway analysis approach, we further show M-MDSC from CoV2+ individuals are enriched in pathways that regulate both innate and adaptive immune control mechanisms. Importantly, the genes in these pathways remain downregulated long after recovery from COVID-19. These support the previous findings that MDSC regulates CD4 and CD8 T cell responses in CoV2 infection (35, 49). To our knowledge, this is the first demonstration of suppression of T cell recall responses by M-MDSC even after recovery from infection.

MDSC expand during various pathological conditions including microbial infection, due to acute or chronic inflammation. Independent studies on laboratory-confirmed CoV2 infected individuals with varying disease severity establish a higher frequency of both CD14+ M- and CD15+ G-MDSC in individuals with severe disease (35, 49–52). Of note, circulating MDSC inversely correlate with T cell count suggesting the contribution of these cells to T cell dysfunction (49, 50). In the present study, we provide evidence of M-MDSC mediated suppression of CoV2 immunogenic antigen-specific T cell responses long after the first exposure to the virus. COVID-19 is associated with massive immune activation, with elevated levels of proinflammatory cytokine IL-6. Therapeutic modulation of IL-6 levels by anti-IL-6 receptor monoclonal antibodies (mAbs) (tocilizumab, sarilumab) and anti-IL6 mAbs reduces the duration and/or severity of COVID-19 (87–90). Longitudinal studies on proinflammatory cytokine trajectory in COVID-19 remain unexplored, in this study, we found elevated levels of IL-6 and IL-8 in CoV2+ individuals even after 5-months of the first detection of infection. However, only IL-6 correlated with the frequency of M-MDSC. To our knowledge, this is the first demonstration of a direct correlation of plasma IL-6 level with circulating frequency of M-MDSC in CoV2+ individuals.

Although the mechanism of IL-8 (CXCL8) mediated M-MDSC expansion is not established, IL-8 facilitates differentiation and subsequent mobilization of MDSC in patients diagnosed with several types of cancers (91, 92). IL-6, through gp130/JAK/STAT pathway, phosphorylates STAT3, which translocates to the nucleus and regulates differentiation, mobilization, and survival of M-MDSC in human and mice disease conditions including COVID-19 (39, 41, 46, 93). An increased gene expression of *STAT3* in M-MDSC of CoV2+ individuals suggests that IL-6-STAT3 is the critical mediator of M-MDSC expansion following CoV2 infection. In our study, *STAT3* expression did not reach significance; this could be due to 1) a small sample size, and 2) lower *STAT3* expression in CoV2+ individuals exhibiting a low level of M-MDSC (**Supplementary Figure 3**). Severe COVID-19 causes a dysregulated myeloid cell compartment resulting in an increase in dysfunctional HLA DR^lo^S100A^hi^ monocytes cells resembling MDSC (80). In contrast, our transcriptomic data showed a trend of lower S100A9/A8 expression in CD14^+^HLA DR^lo^ M-MDSC isolated 5 months after the first detection of CoV2 (**Supplementary Table 2**). Even though the markers differentiating G- and M-MDSC in COVID-19 infection are not well established, S100A9/A8 proteins are majorly expressed by G-MDSC, and our transcriptomic analysis did not show the expression of *CECAM8* (CD66b), a marker exclusively expressed on G-MDSC (28, 31, 44). It is also possible that the downregulation of S100A proteins in our study is due to the control in hyper inflammation over a period of time post-infection. Our findings resemble the transcriptional signature of M-MDSC reported by Kvedaraite *et al*, demonstrating an increased expression of *IFITM2*, *IFITM3*, and lower expression of *CD83* (**Supplementary Table 2**) in cells isolated from CoV2+ individuals as compared to CoV2- controls (94). We show here that the transcriptional signatures of M-MDSC and T cell suppression due to M-MDSC is maintained as long as after 5 months of the first detection of the infection. It appears that aberrant myelopoiesis continues after recovery from COVID-19, and IL-8 and IL-6 remain important mediators for the mobilization and expansion of STAT3 expressing M-MDSC.

STAT3 directly regulates the p47^phox^ subunit of ROS producing NADPH oxidase (NOX2) complex (42, 95). Our finding that M-MDSC isolated from CoV2+ individuals express more *p47^phox^*, and ROS neutralization in M-MDSC depleted cultures did not augment IFNγ production in response to the peptide pool of immune dominant S-antigen is similar to other viral infections (96, 97). Efficient T cell signaling and subsequent immune effector function require proper interaction between T cell receptors (TCR) on APC and CD3 on T cells. ROS contributes to the generation of peroxynitrite that causes nitration of a tyrosine residue at TCR-CD3 complex in CD8^+^ cells, thereby weakening CD8**-**TCR interaction (98, 99). Whether ROS in M-MDSC of CoV2+ individuals also modifies tyrosine or any other amino acid at the TCR-T cell junction remains to be explored. Additionally, amino acid L-arginine is essential for T cell activation and function. Its depletion in the cell culture leads to a rapid decrease in CD3ζ levels, and this is reversed by L-arginine supplementation *in vitro* or arginase (ARG) inhibition when co-culture with ARG-producing cells is used (100–103). The two isoforms ARG1 (encoded by *ARG1*) and ARG2 (encoded by *ARG2*) of the enzyme ARG hydrolyze L-arginine (104–106). We found increased expression of *ARG2* gene in M-MDSC of CoV2+ individuals, and ARG inhibition by nor-NOHA in M-MDSC depleted cultures did not affect IFNγ production support the findings of Falck-Jones et al. demonstrating lower expression of CD3ζ-chain in CD4^+^ and CD8^+^ T cells of COVID-19 patients, and an increased T cell function in PBMC-M-MDSC co-cultures supplemented with L-arginine (35). However, our transcriptomic analysis (**Supplementary Table 2**) did not reveal the expression of *ARG1* gene, and *ARG1* expression in M-MDSC measured by qRT PCR remained undetectable suggesting a dominant role of ARG2 dependent depletion of L-arginine by M-MDSC in our study cohort. A similar prominent role of ARG2 in macrophages has been observed in *Helicobacter pylori* infection, and CD8^+^ T cells from ARG2-deficient mice show superior antitumor activity (107, 108). It is highly like that similar to human dendritic cells and other myeloid cell types, both ARG1 and ARG2 contribute to the immune suppressive function of M-MDSC in CoV2 infection; nonetheless, the relative contribution of the two isoforms to deplete L-arginine might be dependent on the disease stage (109, 110). Here we propose that ROS and Arginase produced by M-MDSC are the mediators of CoV2 antigen-specific T cell suppression.Increasing evidence suggest that both CD4 and CD8 T cell responses are important for COVID-19 outcome and maintenance of CoV2 immunity, even in the absence of humoral response. T cell responses to CoV2 has been shown to recognize epitopes across multiple viral proteins. Similar to the findings of Saini et al. and Tarke *et al*, we also found that CoV2+ individuals produced significant quantities of T_H_1 cytokines in response to the peptide pools of S, N and M proteins (20, 111). Other studies have shown that CoV2 S, N and M, together with non-structural protein (nsp)3, nsp4 and ORF3 are recognized by T cells (12, 19, 20). Although epitope mapping was beyond the scope of this study, and our sample size was small to establish immune dominant antigen for T cell responses in our cohort, we observed cultures stimulated with peptide pool of N antigen produced the maximum quantities of T_H_1 cytokine, and the T cell response was durable up to 5-months. Independent studies have shown that the durability and stability of CoV2 specific T cell responses are maintained from 3 to 12 months post infection (17). Our findings followed the trend previously observed to corresponding peptide pools, and that T cell recognition of multiple epitopes commonly occurs in CoV2 infection. Both G- and M- MDSC have been shown to suppress T cell proliferation and cytokine production during severe and mild CoV2 infection (35, 49, 52). Previous studies of Agrati et al. and Falck-Jones *et al*, show that depleting MDSC from PBMC of CoV2+ individuals increased the T cell cytokine production in response to Staphylococcus enterotoxin B (SEB) polyclonal stimulation, thus establishing that M-MDSC isolated from these individuals had a potent suppressive effect on T cells (35, 49). Here we show M-MDSC inhibits T cell cytokine production in response to CoV2 antigen, thus in an antigen specific manner. Our findings and those of others also show that MDSC isolated from CoV2+ individuals suppress T cell cytokine production *via* arginase and TGFβ dependent mechanisms. Importantly, we provide the evidence that M-MDSC identified in CoV2+ individuals long after recovery from infection are suppressive and functionally active. To our knowledge, this is the first report showing CoV2 antigen-specific immune suppressive activity of M-MDSC, and at a much later time point.

During chronic viral infection, persistent antigen disrupts memory cell development and leading to impairment in T cell function characterized by upregulation of exhaustion (PD-1, TIM-3) and senescence markers (CD57) (69, 112). Similar to other chronic viral infections we observed an increase in CD8^+^ T_EM_ cells expressing PD-1, and CD4^+^ T_EM_ cells expressing CD57 in the peripheral blood of CoV2+ individuals. However, neither these correlate to the frequency of M-MDSC in peripheral blood, nor does depleting M-MDSC from PBMC affect T_EM_ cell subsets (data not shown). Based on our blocking studies (**Figure 5**) and RNA-seq data (**Supplementary Table 2**), in this study, we provide evidence PD-1-related inhibitory pathways do not contribute to M-MDSC mediated immune suppressive function of T cells in CoV2 infection. Even though the expression of PD-1 during the acute and early convalescent phase of CoV2 infection correlates more with activation state rather than functional exhaustion, future studies on understanding the role of these T cell subsets after clinical recovery from the infection will be important (18, 113). Additionally, COVID-19 vaccines are also administered to COVID-19 convalescent individuals. Given that, exhausted T cells lose their potential to differentiate into memory T cells, T cell exhaustion in individuals with past CoV2 infection can impede vaccine-induced T cell memory development.

Antigen presentation is a vital immune process essential for triggering T cell response and is a fundamental element of host defense (114). Classical antigen-presenting cells are of myeloid (macrophages, monocytes, dendritic cells) origin, which in association with MHC class II (for exogenous antigens) and MHC class I (for endogenous antigens) molecules activate CD4^+^ and CD8^+^ T cells, respectively (114). We show here that M-MDSC isolated from CoV2+ individuals suppress T cell cytokine production in an antigen-specific manner. Although we studied IL-2 production, but not other cytokines exclusively produced by CD4^+^ and CD8^+^ T cells, in line with this data, we found the genes *HLA DQB1*, *HLA DQA1*, and *HLA A* critical for antigen presentation are down-regulated in M-MDSC isolated from all CoV2+ individuals. Among the various genes categorized in the KEGG enriched antigen processing and presentation pathway, *CD74*, *HSPA8*, and *PSME2* genes involved in the processing of exogenous antigens exhibited a variable expression pattern among CoV2+ participants (115). Similarly, *PSME2* implicated in immunoproteasome assembly and required for efficient antigen processing of endogenous antigens also exhibited variable expression among CoV2+ participants (116). It appears that other participant-dependent factors may regulate antigen processing in M-MDSC, but inefficient antigen presentation is the dominant factor driving suppressed T cell responses in CoV2+ participants. Another pathway in the KEGG database related to pathogen/antigen uptake and its presentation to T cells is Phagosome. Although the expression of *FCGR3A* responsible for FcR mediated phagocytosis was variable among the CoV2+ participants, genes coding for proteins associated with Phagolysosome Biogenesis (*THBS1*) and microtubule activity of phagosome pathway (*TFRC*) along with *HLA DQB1*, *HLA DQA1*, and *HLA A* were consistently down-regulated (117, 118). Together, these data suggest that CoV2 infection induces long-term changes that negatively influence anti-CoV2 T cell response.

Additionally, compared to CoV2-, multiple pathways related to innate immune signaling were also enriched in M-MDSC of Cov2+ individuals. However, the genes driving these pathways were majorly downregulated. IL-1β-NLRP3 axis has a double-sword function in anti-viral immunity which may facilitate viral eradication but also results in the proinflammatory cell death known as pyroptosis (119). Chemokines CXCL1 and CXCL2 direct the recruitment of various immune effector cells and also activate the NLRP3 inflammasome (120). PI3K/AKT is one of the most critical pathways in innate immune signaling and cell survival. Here we found that genes *IRS2*, *NRG1*, *HBEGF*, and *EREG* regulating AKT signaling are downregulated in M-MDSC isolated from CoV2+ participants long after recovery from infection (121). These data suggest that during recovery from infection M-MDSC may have developed a mechanism to countermeasure hyper inflammation by inhibiting innate immune signaling, but retain their capacity to suppress T cell responses.

### Limitations of Study

The pathological and immunological consequences of M-MDSC after recovery from CoV2 remain unclear. Given the immune regulatory role of M-MDSC in various infections and immunological abnormalities, it is highly likely that M-MDSC in individuals with critical COVID-19 illness contributes to immunosuppression (35, 49–52). While our study design provides robust and reproducible results suggesting an expansion of M-MDSC even after 5-months of the first report of infection, which exhibits the dual function of suppressing T cells responses and downregulated inflammatory responses, the small sample size in our study is a major limitation and reason for some findings unable to reach statistical significance. Thus, other mechanisms driving the response of M-MDSC, such as genetics, comorbidities, age, gender, or initial viral load remain unexplored. Due to the increased rate of vaccination in this community, the identification of non-vaccinated participants with a history of CoV2 infection, and non-vaccinated uninfected controls became a challenge. Thus this study was performed on a small cohort of non-vaccinated CoV2- and CoV2+ individuals. We performed RNA-seq studies in two batches to compare the global gene profile of M-MDSC in CoV2- and CoV2+ individuals; however, we observed a huge batch effect (**Supplemental Figures 2**, **4**) with less coverage of reads across human transcriptome in Batch 2. This resulted in the identification of limited numbers of DEGs (12 DEGs with FDR <0.05) in M-MDSC from CoV2+ individuals, and the fold change expression of many genes (*CSTA*, *SELL* (CD62L), *S100A8/A9*, *FCN1*, *DUSP6*) important for characterization and function of M-MDSC did not reach statistical significance. Additionally, two CoV2- samples were outliers and excluded from the analysis. Despite these limitations, our study clearly demonstrates that M-MDSC retains their capacity to suppress T cell function even after recovery from CoV2 infection. Indeed, future studies performed on large cohorts of individuals receiving the COVID-19 vaccine will be important to establish the effect of M-MDSC on long-term vaccine efficacy.

## Conclusion

Collectively, our *ex vivo* and *in vitro* data establish that CoV2 infected individuals have an increased quantity of M-MDSC even after 5-months of the first report of infection. Importantly, we show that M-MDSC controls T cell recall responses to peptides of major antigenic proteins of the virus. In addition, we show transcriptional changes in M-MDSC of CoV2 + individuals as compared to those of CoV2- individuals. Our findings suggest that M-MDSC are important regulators of immune responses, and provide attractive targets to augment vaccine responses. Our ongoing studies are centered to study differences in M-MDSC population in response to various CoV2 vaccines. An improved understanding of MDSC biology in CoV2 infection can contribute to novel approaches to prolong durability of vaccine response and/or restore normal immune function.

## Data Availability Statement

The datasets presented in this study can be found in online repositories. The name of the repository and accession number can be found below: (https://www.ncbi.nlm.nih.gov/geo/query/acc.cgi?acc=GSE199286).

## Ethics Statement

The studies involving human participants were reviewed and approved by Institutional review board of the University of Georgia, Athens. The patients/participants provided their written informed consent to participate in this study.

## Author Contributions

AG developed the study, performed experiments and analyzed the data; KM performed the experiments; RU and LS performed the preliminary analysis of RNA-seq data; NBB analyzed RNA-seq data; AG and NBB wrote the manuscript.

## Funding

AG was supported in part by AI127132 from the National Institute of Allergy and Infectious Diseases (NIAID), IUSSTF/VN-COVID/011/2020 from INDO-US Science and Technology Forum, and The University of Georgia Research Foundation. A part of this work was supported by the National Center for Advancing Translational Sciences of the National Institutes of Health under Award Number UL1TR002378. NB-B was supported in part by 1I01 BX005285 from the Office of Research and Development, Veterans Health Administration and R56 AI157755 (NIH/NIAID). LS and RX were supported by LS’s startup funds from the University of Georgia Office of Research.

## Author Disclaimer

The content is solely the responsibility of the authors and does not necessarily represent the official views of the National Institutes of Health.

## Conflict of Interest

The authors declare that the research was conducted in the absence of any commercial or financial relationships that could be construed as a potential conflict of interest.

## Publisher’s Note

All claims expressed in this article are solely those of the authors and do not necessarily represent those of their affiliated organizations, or those of the publisher, the editors and the reviewers. Any product that may be evaluated in this article, or claim that may be made by its manufacturer, is not guaranteed or endorsed by the publisher.

## Supplementary Material

Supplementary Table 1 Primers used in the study for targeted gene expression: M-MDSC were sorted by flow cytometry and stored in Trizol. RNA was isolated and gene expression of genes was determined by qRT PCR as detailed in Methods. The list provides the sequences and annealing temperature of the genes studied in the study.

Supplementary Table 2 Differential gene expression analysis between CoV2+ and CoV2- groups. A total of 9,217 genes survived our filtering criteria. Genes in the table are sorted first by FDR, then by nominal *p*-value from least to greatest, and then alphabetically by gene ENSEMBL_ID. Counts per million reads for each gene are available for all samples included in the analysis. *LogFC*, log_2_ transformed values of fold changes in gene expression between CoV2+ and CoV2- samples; *FC*, fold changes in gene expression between CoV2+ and CoV2- samples; *FDR*, false discovery rate corrected *p*-value; *cmp*, counts per million.

Supplementary Table 3 Pathways enriched for DEGs identified between CoV2+ and CoV2- groups (nominal *p*-value <0.05). Pathway enrichment analysis was conducted using the Database for Annotation, Visualization and Integrated Discovery (DAVID) v6.8 and KEGG and Reactome databases. Pathways with nominal *p*-value <0.1 were considered significant. Pathways identified from both databases were combined and sorted by the nominal *p*-value.

## References

[1] Zhou F Yu T Du R Fan G Liu Y Liu Z. Clinical Course and Risk Factors for Mortality of Adult Inpatients With COVID-19 in Wuhan, China: A Retrospective Cohort Study. Lancet (2020) 395:1054–62. doi: 10.1016/S0140-6736(20)30566-3 PMC7270627 32171076

[2] Del Valle DM Kim-Schulze S Huang HH Beckmann ND Nirenberg S Wang B. An Inflammatory Cytokine Signature Predicts COVID-19 Severity and Survival. Nat Med (2020) 26:1636–43. doi: 10.1038/s41591-020-1051-9 PMC7869028 32839624

[3] Chen G Wu D Guo W Cao Y Huang D Wang H. Clinical and Immunological Features of Severe and Moderate Coronavirus Disease 2019. J Clin Invest (2020) 130:2620–9. doi: 10.1172/JCI137244 PMC7190990 32217835

[4] Gao Y Li T Han M Li X Wu D Xu Y. Diagnostic Utility of Clinical Laboratory Data Determinations for Patients With the Severe COVID-19 (2020). J Med Virol 92(7):791–6. doi: 10.1002/jmv.25770 PMC7228247 32181911

[5] Huang C Wang Y Li X Ren L Zhao J Hu Y. Clinical Features of Patients Infected With 2019 Novel Coronavirus in Wuhan, China. Lancet (2020) 395:497–506. doi: 10.1016/S0140-6736(20)30183-5 31986264 PMC7159299

[6] Ragab D Salah Eldin H Taeimah M Khattab R Salem R. The COVID-19 Cytokine Storm; What We Know So Far (2020). Front Immunol 11:1446. doi: 10.3389/fimmu.2020.01446 32612617 PMC7308649

[7] Wang Z Yang X Zhou Y Sun J Liu X Zhang J. COVID-19 Severity Correlates With Weaker T-Cell Immunity, Hypercytokinemia, and Lung Epithelium Injury. Am J Respir Crit Care Med (2020) 202:606–10. doi: 10.1164/rccm.202005-1701LE PMC7427397 32608999

[8] Zheng M Gao Y Wang G Song G Liu S Sun D. Functional Exhaustion of Antiviral Lymphocytes in COVID-19 Patients (2020). Cell Mol Immunol 17:533–5. doi: 10.1038/s41423-020-0402-2 PMC7091858 32203188

[9] Jiang M Guo Y Luo Q Huang Z Zhao R Liu S. T-Cell Subset Counts in Peripheral Blood Can Be Used as Discriminatory Biomarkers for Diagnosis and Severity Prediction of Coronavirus Disease 2019. J Infect Dis (2020)222:198–202. doi: 10.1093/infdis/jiaa252 32379887 PMC7239156

[10] Kang CK Han GC Kim M Kim G Shin HM Song KH. Aberrant Hyperactivation of Cytotoxic T-Cell as a Potential Determinant of COVID-19 Severity. Int J Infect Dis (2020) 97:313–21. doi: 10.1016/j.ijid.2020.05.106 PMC7261468 32492530

[11] de Candia P Prattichizzo F Garavelli S Matarese G. T Cells: Warriors of SARS-CoV-2 Infection. Trends Immunol (2021) 42:18–30. doi: 10.1016/j.it.2020.11.002 33277181 PMC7664351

[12] Grifoni A Weiskopf D Ramirez SI Mateus J Dan JM Moderbacher CR. Targets of T Cell Responses to SARS-CoV-2 Coronavirus in Humans With COVID-19 Disease and Unexposed Individuals. Cell (2020) 181:1489–1501.e15. doi: 10.1016/j.cell.2020.05.015 32473127 PMC7237901

[13] Liu R Wang Y Li J Han H Xia Z Liu F. Decreased T Cell Populations Contribute to the Increased Severity of COVID-19. Clin Chim Acta (2020) 508:110–4. doi: 10.1016/j.cca.2020.05.019 PMC7219428 32405080

[14] Liu Z Long W Tu M Chen S Huang Y Wang S. Lymphocyte Subset (CD4+, CD8+) Counts Reflect the Severity of Infection and Predict the Clinical Outcomes in Patients With COVID-19. J Infect (2020) 81:318–56. doi: 10.1016/j.jinf.2020.03.054 PMC7151318 32283159

[15] Lafon E Diem G Witting C Zaderer V Bellmann-Weiler RM Reindl M. Potent SARS-CoV-2-Specific T Cell Immunity and Low Anaphylatoxin Levels Correlate With Mild Disease Progression in COVID-19 Patients. Front Immunol (2021) 12:684014. doi: 10.3389/fimmu.2021.684014 34194438 PMC8237940

[16] Le Bert N Tan AT Kunasegaran K Tham CYL Hafezi M Chia A. SARS-CoV-2-Specific T Cell Immunity in Cases of COVID-19 and SARS, and Uninfected Controls. Nature (2020) 584:457–62. doi: 10.1038/s41586-020-2550-z 32668444

[17] Lu Z Laing ED Pena DaMata J Pohida K Tso MS Samuels EC. Durability of SARS-CoV-2-Specific T-Cell Responses at 12 Months Postinfection. J Infect Dis (2021) 224:2010–9. doi: 10.1093/infdis/jiab543 PMC8672777 34673956

[18] Sekine T Perez-Potti A Rivera-Ballesteros O Stralin K Gorin JB Olsson A. Robust T Cell Immunity in Convalescent Individuals With Asymptomatic or Mild COVID-19. Cell (2020) 183:158–168.e14. doi: 10.1016/j.cell.2020.08.017 32979941 PMC7427556

[19] Nelde A Bilich T Heitmann JS Maringer Y Salih HR Roerden M. SARS-CoV-2-Derived Peptides Define Heterologous and COVID-19-Induced T Cell Recognition. Nat Immunol (2021) 22:74–85. doi: 10.1038/s41590-020-00808-x 32999467

[20] Tarke A Sidney J Kidd CK Dan JM Ramirez SI Yu ED. Comprehensive Analysis of T Cell Immunodominance and Immunoprevalence of SARS-CoV-2 Epitopes in COVID-19 Cases. Cell Rep Med (2021) 2:100204. doi: 10.1016/j.xcrm.2021.100204 33521695 PMC7837622

[21] Jung JH Rha MS Sa M Choi HK Jeon JH Seok H. SARS-CoV-2-Specific T Cell Memory Is Sustained in COVID-19 Convalescent Patients for 10 Months With Successful Development of Stem Cell-Like Memory T Cells. Nat Commun (2021) 12:4043. doi: 10.1038/s41467-021-24377-1 34193870 PMC8245549

[22] Bonifacius A Tischer-Zimmermann S Dragon AC Gussarow D Vogel A Krettek U. COVID-19 Immune Signatures Reveal Stable Antiviral T Cell Function Despite Declining Humoral Responses. Immunity (2021) 54:340–354.e6. doi: 10.1016/j.immuni.2021.01.008 33567252 PMC7871825

[23] Breton G Mendoza P Hagglof T Oliveira TY Schaefer-Babajew D Gaebler C. Persistent Cellular Immunity to SARS-CoV-2 Infection. J Exp Med (2021) 218(4):e20202515. doi: 10.1084/jem.20202515 33533915 PMC7845919

[24] Dan JM Mateus J Kato Y Hastie KM Yu ED Faliti CE. Immunological Memory to SARS-CoV-2 Assessed for Up to 8 Months After Infection. Science (2021) 371(6529):eabf4063. doi: 10.1126/science.abf4063 33408181 PMC7919858

[25] Le Bert N Clapham HE Tan AT Chia WN Tham CYL Lim JM. Highly Functional Virus-Specific Cellular Immune Response in Asymptomatic SARS-CoV-2 Infection. J Exp Med (2021) 218:e20202617. doi: 10.1084/jem.20202617 33646265 PMC7927662

[26] Reynolds CJ Swadling L Gibbons JM Pade C Jensen MP Diniz MO. Discordant Neutralizing Antibody and T Cell Responses in Asymptomatic and Mild SARS-CoV-2 Infection. Sci Immunol (2020) 5(54):eabf3698. doi: 10.1126/sciimmunol.abf3698 33361161 PMC8101131

[27] Rodda LB Netland J Shehata L Pruner KB Morawski PA Thouvenel CD. Functional SARS-CoV-2-Specific Immune Memory Persists After Mild COVID-19. Cell (2021) 184:169–183.e17. doi: 10.1016/j.cell.2020.11.029 33296701 PMC7682481

[28] Bronte V Brandau S Chen SH Colombo MP Frey AB Greten TF. Recommendations for Myeloid-Derived Suppressor Cell Nomenclature and Characterization Standards. Nat Commun (2016) 7:12150. doi: 10.1038/ncomms12150 27381735 PMC4935811

[29] Delano MJ Scumpia PO Weinstein JS Coco D Nagaraj S Kelly-Scumpia KM. MyD88-Dependent Expansion of an Immature GR-1(+)CD11b(+) Population Induces T Cell Suppression and Th2 Polarization in Sepsis. J Exp Med (2007) 204:1463–74. doi: 10.1084/jem.20062602 PMC2118626 17548519

[30] Elkabets M Ribeiro VS Dinarello CA Ostrand-Rosenberg S Di Santo JP Apte RN. IL-1beta Regulates a Novel Myeloid-Derived Suppressor Cell Subset That Impairs NK Cell Development and Function. Eur J Immunol (2010) 40:3347–57. doi: 10.1002/eji.201041037 PMC3373225 21110318

[31] Gabrilovich DI Nagaraj S. Myeloid-Derived Suppressor Cells as Regulators of the Immune System. Nat Rev Immunol (2009) 9:162–74. doi: 10.1038/nri2506 PMC2828349 19197294

[32] Garg A Spector SA. HIV Type 1 Gp120-Induced Expansion of Myeloid Derived Suppressor Cells is Dependent on Interleukin 6 and Suppresses Immunity. J Infect Dis (2014) 209:441–51. doi: 10.1093/infdis/jit469 PMC3883171 23999600

[33] Hongo D Tang X Baker J Engleman EG Strober S. Requirement for Interactions of Natural Killer T Cells and Myeloid-Derived Suppressor Cells for Transplantation Tolerance. Am J Transplant (2014) 14:2467–77. doi: 10.1111/ajt.12914 PMC4205183 25311657

[34] Li Y Tu Z Qian S Fung JJ Markowitz SD Kusner LL. Myeloid-Derived Suppressor Cells as a Potential Therapy for Experimental Autoimmune Myasthenia Gravis. J Immunol (2014) 193:2127–34. doi: 10.4049/jimmunol.1400857 PMC4784709 25057008

[35] Falck-Jones S Vangeti S Yu M Falck-Jones R Cagigi A Badolati I. Functional Monocytic Myeloid-Derived Suppressor Cells Increase in Blood But Not Airways and Predict COVID-19 Severity. J Clin Invest (2021) 131(6):e144734. doi: 10.1172/JCI144734 PMC7954608 33492309

[36] Koushki K Salemi M Miri SM Arjeini Y Keshavarz M Ghaemi A. Role of Myeloid-Derived Suppressor Cells in Viral Respiratory Infections; Hints for Discovering Therapeutic Targets for COVID-19. BioMed Pharmacother (2021) 144:112346. doi: 10.1016/j.biopha.2021.112346 34678727 PMC8516725

[37] Jeisy-Scott V Davis WG Patel JR Bowzard JB Shieh WJ Zaki SR. Increased MDSC Accumulation and Th2 Biased Response to Influenza A Virus Infection in the Absence of TLR7 in Mice. PloS One (2011) 6:e25242. doi: 10.1371/journal.pone.0025242 21966467 PMC3179470

[38] Filipazzi P Valenti R Huber V Pilla L Canese P Iero M. Identification of a New Subset of Myeloid Suppressor Cells in Peripheral Blood of Melanoma Patients With Modulation by a Granulocyte-Macrophage Colony-Stimulation Factor-Based Antitumor Vaccine. J Clin Oncol (2007) 25:2546–53. doi: 10.1200/JCO.2006.08.5829 17577033

[39] Poschke I Mougiakakos D Hansson J Masucci GV Kiessling R. Immature Immunosuppressive CD14+HLA-DR-/Low Cells in Melanoma Patients are Stat3hi and Overexpress CD80, CD83, and DC-Sign. Cancer Res (2010) 70:4335–45. doi: 10.1158/0008-5472.CAN-09-3767 20484028

[40] Youn JI Nagaraj S Collazo M Gabrilovich DI. Subsets of Myeloid-Derived Suppressor Cells in Tumor-Bearing Mice. J Immunol (2008) 181:5791–802. doi: 10.4049/jimmunol.181.8.5791 PMC2575748 18832739

[41] Condamine T Gabrilovich DI. Molecular Mechanisms Regulating Myeloid-Derived Suppressor Cell Differentiation and Function. Trends Immunol (2011) 32:19–25. doi: 10.1016/j.it.2010.10.002 21067974 PMC3053028

[42] Corzo CA Cotter MJ Cheng P Cheng F Kusmartsev S Sotomayor E. Mechanism Regulating Reactive Oxygen Species in Tumor-Induced Myeloid-Derived Suppressor Cells. J Immunol (2009) 182:5693–701. doi: 10.4049/jimmunol.0900092 PMC2833019 19380816

[43] Lechner MG Liebertz DJ Epstein AL. Characterization of Cytokine-Induced Myeloid-Derived Suppressor Cells From Normal Human Peripheral Blood Mononuclear Cells. J Immunol (2010) 185:2273–84. doi: 10.4049/jimmunol.1000901 PMC2923483 20644162

[44] Lechner MG Megiel C Russell SM Bingham B Arger N Woo T. Functional Characterization of Human Cd33+ and Cd11b+ Myeloid-Derived Suppressor Cell Subsets Induced From Peripheral Blood Mononuclear Cells Co-Cultured With a Diverse Set of Human Tumor Cell Lines. J Transl Med (2011) 9:90. doi: 10.1186/1479-5876-9-90 21658270 PMC3128058

[45] Rodriguez PC Ernstoff MS Hernandez C Atkins M Zabaleta J Sierra R. Arginase I-Producing Myeloid-Derived Suppressor Cells in Renal Cell Carcinoma Are a Subpopulation of Activated Granulocytes. Cancer Res (2009) 69:1553–60. doi: 10.1158/0008-5472.CAN-08-1921 PMC2900845 19201693

[46] Chalmin F Ladoire S Mignot G Vincent J Bruchard M Remy-Martin JP. Membrane-Associated Hsp72 From Tumor-Derived Exosomes Mediates STAT3-Dependent Immunosuppressive Function of Mouse and Human Myeloid-Derived Suppressor Cells. J Clin Invest (2010) 120:457–71. doi: 10.1172/JCI40483 PMC2810085 20093776

[47] Finke J Ko J Rini B Rayman P Ireland J Cohen P. MDSC as a Mechanism of Tumor Escape From Sunitinib Mediated Anti-Angiogenic Therapy. Int Immunopharmacol (2011) 11:856–61. doi: 10.1016/j.intimp.2011.01.030 PMC3109226 21315783

[48] Nefedova Y Huang M Kusmartsev S Bhattacharya R Cheng P Salup R. Hyperactivation of STAT3 is Involved in Abnormal Differentiation of Dendritic Cells in Cancer. J Immunol (2004) 172:464–74. doi: 10.4049/jimmunol.172.1.464 14688356

[49] Agrati C Sacchi A Bordoni V Cimini E Notari S Grassi G. Expansion of Myeloid-Derived Suppressor Cells in Patients With Severe Coronavirus Disease (COVID-19). Cell Death Differ (2020) 27:3196–207. doi: 10.1038/s41418-020-0572-6 PMC7278239 32514047

[50] Reizine F Lesouhaitier M Gregoire M Pinceaux K Gacouin A Maamar A. SARS-CoV-2-Induced ARDS Associates With MDSC Expansion, Lymphocyte Dysfunction, and Arginine Shortage. J Clin Immunol (2021) 41:515–25. doi: 10.1007/s10875-020-00920-5 PMC7775842 33387156

[51] Rowlands M Segal F Hartl D. Myeloid-Derived Suppressor Cells as a Potential Biomarker and Therapeutic Target in COVID-19. Front Immunol (2021) 12:697405. doi: 10.3389/fimmu.2021.697405 34220859 PMC8250151

[52] Sacchi A Grassi G Bordoni V Lorenzini P Cimini E Casetti R. Early Expansion of Myeloid-Derived Suppressor Cells Inhibits SARS-CoV-2 Specific T-Cell Response and may Predict Fatal COVID-19 Outcome. Cell Death Dis (2020) 11:921. doi: 10.1038/s41419-020-03125-1 33110074 PMC7590570

[53] Vitte J Diallo AB Boumaza A Lopez A Michel M Allardet-Servent J. A Granulocytic Signature Identifies COVID-19 and Its Severity. J Infect Dis (2020) 222:1985–96. doi: 10.1093/infdis/jiaa591 PMC7543529 32941618

[54] Namdev P Patel S Sparling B Garg A. Monocytic-Myeloid Derived Suppressor Cells of HIV-Infected Individuals With Viral Suppression Exhibit Suppressed Innate Immunity to Mycobacterium Tuberculosis. Front Immunol (2021) 12:647019. doi: 10.3389/fimmu.2021.647019 33995365 PMC8113814

[55] Pfaffl MW. A New Mathematical Model for Relative Quantification in Real-Time RT-PCR. Nucleic Acids Res (2001) 29:e45. doi: 10.1093/nar/29.9.e45 11328886 PMC55695

[56] Martin M. Cutadapt Removes Adapter Sequences From High-Throughput Sequencing Reads. EMBnet (2011) 17:3. doi: 10.14806/ej.17.1.200

[57] Kim D Langmead B Salzberg SL. HISAT: A Fast Spliced Aligner With Low Memory Requirements. Nat Methods (2015) 12:357–60. doi: 10.1038/nmeth.3317 PMC4655817 25751142

[58] Harrow J Frankish A Gonzalez JM Tapanari E Diekhans M Kokocinski F. GENCODE: The Reference Human Genome Annotation for The ENCODE Project. Genome Res (2012) 22:1760–74. doi: 10.1101/gr.135350.111 PMC3431492 22955987

[59] Anders S Pyl PT Huber W. HTSeq–a Python Framework to Work With High-Throughput Sequencing Data. Bioinformatics (2015) 31:166–9. doi: 10.1093/bioinformatics/btu638 PMC4287950 25260700

[60] Robinson MD McCarthy DJ Smyth GK. Edger: A Bioconductor Package for Differential Expression Analysis of Digital Gene Expression Data. Bioinformatics (2010) 26:139–40. doi: 10.1093/bioinformatics/btp616 PMC2796818 19910308

[61] Zhang Y Parmigiani G Johnson WE. ComBat-Seq: Batch Effect Adjustment for RNA-Seq Count Data. NAR Genom Bioinform (2020) 2:lqaa078. doi: 10.1093/nargab/lqaa078 33015620 PMC7518324

[62] Hochberg Y Benjamini Y. Controlling the False Discovery Rate: A Practical and Powerful Approach to Multiple Testing. J R Stat Soc B MET (1995) 57:12.

[63] DennisGJr.ShermanBTHosackDAYangJGaoWLaneHC. DAVID: Database for Annotation, Visualization, and Integrated Discovery. Genome Biol (2003) 4:P3. doi: 10.1186/gb-2003-4-5-p3 12734009

[64] Huang da W Sherman BT Lempicki RA. Bioinformatics Enrichment Tools: Paths Toward the Comprehensive Functional Analysis of Large Gene Lists. Nucleic Acids Res (2009) 37:1–13. doi: 10.1093/nar/gkn923 19033363 PMC2615629

[65] Kanehisa M Goto S. KEGG: Kyoto Encyclopedia of Genes and Genomes. Nucleic Acids Res (2000) 28:27–30. doi: 10.1093/nar/28.1.27 10592173 PMC102409

[66] Ogata H Goto S Sato K Fujibuchi W Bono H Kanehisa M. KEGG: Kyoto Encyclopedia of Genes and Genomes. Nucleic Acids Res (1999) 27:29–34. doi: 10.1093/nar/27.1.29 9847135 PMC148090

[67] Fabregat A Sidiropoulos K Garapati P Gillespie M Hausmann K Haw R. The Reactome Pathway Knowledgebase. Nucleic Acids Res (2016) 44:D481–7. doi: 10.1093/nar/gkv1351 PMC4702931 26656494

[68] Singh R Chakraborty M Gautam A Roy SK Halder I Barber J. Residual Immune Activation in HIV-Infected Individuals Expands Monocytic-Myeloid Derived Suppressor Cells. Cell Immunol (2021) 362:104304. doi: 10.1016/j.cellimm.2021.104304 33610024 PMC8112256

[69] Larbi A Fulop T. From “Truly Naive” to “Exhausted Senescent” T Cells: When Markers Predict Functionality. Cytometry A (2014) 85:25–35. doi: 10.1002/cyto.a.22351 24124072

[70] Sallusto F Geginat J Lanzavecchia A. Central Memory and Effector Memory T Cell Subsets: Function, Generation, and Maintenance. Annu Rev Immunol (2004) 22:745–63. doi: 10.1146/annurev.immunol.22.012703.104702 15032595

[71] Sathaliyawala T Kubota M Yudanin N Turner D Camp P Thome JJ. Distribution and Compartmentalization of Human Circulating and Tissue-Resident Memory T Cell Subsets. Immunity (2013) 38:187–97. doi: 10.1016/j.immuni.2012.09.020 PMC3557604 23260195

[72] Surh CD Sprent J. Homeostasis of Naive and Memory T Cells. Immunity (2008) 29:848–62. doi: 10.1016/j.immuni.2008.11.002 19100699

[73] Rha MS Kim AR Shin EC. SARS-CoV-2-Specific T Cell Responses in Patients With COVID-19 and Unexposed Individuals. Immune Netw (2021) 21:e2. doi: 10.4110/in.2021.21.e2 33728095 PMC7937509

[74] Moss P. The T Cell Immune Response Against SARS-CoV-2. Nat Immunol (2022) 23:186–93. doi: 10.1038/s41590-021-01122-w 35105982

[75] Rha MS Shin EC. Activation or Exhaustion of CD8(+) T Cells in Patients With COVID-19. Cell Mol Immunol (2021) 18:2325–33. doi: 10.1038/s41423-021-00750-4 PMC8374113 34413488

[76] Garg A Trout R Spector SA. Human Immunodeficiency Virus Type-1 Myeloid Derived Suppressor Cells Inhibit Cytomegalovirus Inflammation Through Interleukin-27 and B7-H4. Sci Rep (2017) 7:44485. doi: 10.1038/srep44485 28338007 PMC5364511

[77] Liu H Lin S Ao X Gong X Liu C Xu D. Meta-Analysis of Transcriptome Datasets: An Alternative Method to Study IL-6 Regulation in Coronavirus Disease 2019. Comput Struct Biotechnol J (2021) 19:767–76. doi: 10.1016/j.csbj.2020.12.010 PMC7836900 33520118

[78] Zhang Y Wang S Xia H Guo J He K Huang C. Identification of Monocytes Associated With Severe COVID-19 in the PBMCs of Severely Infected Patients Through Single-Cell Transcriptome Sequencing, (Beijing: Engineering) (2021). doi: 10.1016/j.eng.2021.05.009 PMC8196473 34150352

[79] Zamanian-Azodi M Arjmand B Razzaghi M Rezaei Tavirani M Ahmadzadeh A Rostaminejad M. Platelet and Haemostasis are the Main Targets in Severe Cases of COVID-19 Infection; a System Biology Study. Arch Acad Emerg Med (2021) 9:e27. doi: 10.22037/aaem.v9i1.1108 34027422 PMC8126352

[80] Schulte-Schrepping J Reusch N Paclik D Bassler K Schlickeiser S Zhang B. Severe COVID-19 Is Marked by a Dysregulated Myeloid Cell Compartment. Cell (2020) 182:1419–1440.e23. doi: 10.1016/j.cell.2020.08.001 32810438 PMC7405822

[81] Zhou Z Ren L Zhang L Zhong J Xiao Y Jia Z. Heightened Innate Immune Responses in the Respiratory Tract of COVID-19 Patients. Cell Host Microbe (2020) 27:883–890.e2. doi: 10.1016/j.chom.2020.04.017 32407669 PMC7196896

[82] Available at: https://www.cdc.gov/coronavirus/2019-ncov/vaccines/How-Do-I-Get-a-COVID-19-Vaccine.html?s_cid=10513:%2Bcovid%20%2Bvaccines%20%2Bavailable:sem.b:p:RG:GM:gen:PTN:FY21.

[83] Self WH Tenforde MW Rhoads JP Gaglani M Ginde AA Douin DJ. Vaccines in Preventing COVID-19 Hospitalizations Among Adults Without Immunocompromising Conditions - United States, March-August 2021. MMWR Morb Mortal Wkly Rep (2021) 70:1337–43. doi: 10.15585/mmwr.mm7038e1 PMC8459899 34555004

[84] Zheng C Shao W Chen X Zhang B Wang G Zhang W. Real-World Effectiveness of COVID-19 Vaccines: A Literature Review and Meta-Analysis. Int J Infect Dis (2022) 114:252–60. doi: 10.1016/j.ijid.2021.11.009 PMC8595975 34800687

[85] Feikin DR Higdon MM Abu-Raddad LJ Andrews N Araos R Goldberg Y. Duration of Effectiveness of Vaccines Against SARS-CoV-2 Infection and COVID-19 Disease: Results of a Systematic Review and Meta-Regression. Lancet (2022) 399(10328):P924–944. doi: 10.1016/S0140-6736(22)00152-0 PMC8863502 35202601

[86] Mateus J Dan JM Zhang Z Rydyznski Moderbacher C Lammers M Goodwin B. Low-Dose mRNA-1273 COVID-19 Vaccine Generates Durable Memory Enhanced by Cross-Reactive T Cells. Science (2021) 374:eabj9853. doi: 10.1126/science.abj9853 34519540 PMC8542617

[87] R.C. Group. Tocilizumab in Patients Admitted to Hospital With COVID-19 (RECOVERY): A Randomised, Controlled, Open-Label, Platform Trial. Lancet (2021) 397:1637–45. doi: 10.1016/S0140-6736(21)00676-0 PMC8084355 33933206

[88] Investigators R-C Gordon AC Mouncey PR Al-Beidh F Rowan KM Nichol AD. Interleukin-6 Receptor Antagonists in Critically Ill Patients With Covid-19. N Engl J Med (2021) 384:1491–502. doi: 10.1056/NEJMoa2100433 PMC7953461 33631065

[89] Lescure FX Honda H Fowler RA Lazar JS Shi G Wung P. Sarilumab, Sarilumab in Patients Admitted to Hospital With Severe or Critical COVID-19: A Randomised, Double-Blind, Placebo-Controlled, Phase 3 Trial. Lancet Respir Med (2021) 9:522–32. doi: 10.1016/S2213-2600(21)00099-0 PMC8078879 33676590

[90] Stone JH Frigault MJ Serling-Boyd NJ Fernandes AD Harvey L Foulkes AS. Investigators, Efficacy of Tocilizumab in Patients Hospitalized With Covid-19. N Engl J Med (2020) 383:2333–44. doi: 10.1056/NEJMoa2028836 PMC7646626 33085857

[91] Alfaro C Teijeira A Onate C Perez G Sanmamed MF Andueza MP. Tumor-Produced Interleukin-8 Attracts Human Myeloid-Derived Suppressor Cells and Elicits Extrusion of Neutrophil Extracellular Traps (NETs). Clin Cancer Res (2016) 22:3924–36. doi: 10.1158/1078-0432.CCR-15-2463 26957562

[92] Poschke I Kiessling R. On the Armament and Appearances of Human Myeloid-Derived Suppressor Cells. Clin Immunol (2012) 144:250–68. doi: 10.1016/j.clim.2012.06.003 22858650

[93] Horvath CM. The Jak-STAT Pathway Stimulated by Interleukin. Sci STKE (2004) 6:tr9. doi: 10.1126/stke.2602004tr9 15561981

[94] Kvedaraite E Hertwig L Sinha I Ponzetta A Hed Myrberg I Lourda M. Major Alterations in the Mononuclear Phagocyte Landscape Associated With COVID-19 Severity. Proc Natl Acad Sci U S A 118 (2021) 118(6):e2018587118. doi: 10.1073/pnas.2018587118 PMC8017719 33479167

[95] Sareila O Kelkka T Pizzolla A Hultqvist M Holmdahl R. NOX2 Complex-Derived ROS as Immune Regulators. Antioxid Redox Signal (2011) 15:2197–208. doi: 10.1089/ars.2010.3635 20919938

[96] Gama L Shirk EN Russell JN Carvalho KI Li M Queen SE. Expansion of a Subset of CD14highCD16negCCR2low/neg Monocytes Functionally Similar to Myeloid-Derived Suppressor Cells During SIV and HIV Infection. J Leukoc Biol (2012) 91:803–16. doi: 10.1189/jlb.1111579 PMC3336772 22368280

[97] Schols D De Clercq E. Human Immunodeficiency Virus Type 1 Gp120 Induces Anergy in Human Peripheral Blood Lymphocytes by Inducing Interleukin-10 Production. J Virol (1996) 70:4953–60. doi: 10.1128/jvi.70.8.4953-4960.1996 PMC190447 8764000

[98] Kusmartsev S Nefedova Y Yoder D Gabrilovich DI. Antigen-Specific Inhibition of CD8+ T Cell Response by Immature Myeloid Cells in Cancer is Mediated by Reactive Oxygen Species. J Immunol (2004) 172:989–99. doi: 10.4049/jimmunol.172.2.989 14707072

[99] Nagaraj S Gupta K Pisarev V Kinarsky L Sherman S Kang L. Altered Recognition of Antigen Is a Mechanism of CD8+ T Cell Tolerance in Cancer. Nat Med (2007) 13:828–35. doi: 10.1038/nm1609 PMC2135607 17603493

[100] Rodriguez PC Zea AH Culotta KS Zabaleta J Ochoa JB Ochoa AC. Regulation of T Cell Receptor CD3zeta Chain Expression by L-Arginine. J Biol Chem (2002) 277:21123–9. doi: 10.1074/jbc.M110675200 11950832

[101] Taheri F Ochoa JB Faghiri Z Culotta K Park HJ Lan MS. L-Arginine Regulates the Expression of the T-Cell Receptor Zeta Chain (CD3zeta) in Jurkat Cells. Clin Cancer Res (2001) 7(3 Suppl):958s–65s. 11300497

[102] Rodriguez PC Quiceno DG Zabaleta J Ortiz B Zea AH Piazuelo MB. Arginase I Production in the Tumor Microenvironment by Mature Myeloid Cells Inhibits T-Cell Receptor Expression and Antigen-Specific T-Cell Responses. Cancer Res (2004) 64:5839–49. doi: 10.1158/0008-5472.CAN-04-0465 15313928

[103] Rodriguez PC Zea AH DeSalvo J Culotta KS Zabaleta J Quiceno DG. L-Arginine Consumption by Macrophages Modulates the Expression of CD3 Zeta Chain in T Lymphocytes. J Immunol (2003) 171:1232–9. doi: 10.4049/jimmunol.171.3.1232 12874210

[104] Chen F Lucas R Fulton D. The Subcellular Compartmentalization of Arginine Metabolizing Enzymes and Their Role in Endothelial Dysfunction. Front Immunol (2013) 4:184. doi: 10.3389/fimmu.2013.00184 23847624 PMC3705211

[105] Dizikes GJ Grody WW Kern RM Cederbaum SD. Isolation of Human Liver Arginase cDNA and Demonstration of Nonhomology Between the Two Human Arginase Genes. Biochem Biophys Res Commun (1986) 141:53–9. doi: 10.1016/S0006-291X(86)80333-3 3801008

[106] Grzywa TM Sosnowska A Matryba P Rydzynska Z Jasinski M Nowis D. Myeloid Cell-Derived Arginase in Cancer Immune Response. Front Immunol (2020) 11:938. doi: 10.3389/fimmu.2020.00938 32499785 PMC7242730

[107] Marti i Lindez AA Dunand-Sauthier I Conti M Gobet F Nunez N Hannich JT. Mitochondrial Arginase-2 Is a Cell-Autonomous Regulator of CD8+ T Cell Function and Antitumor Efficacy. JCI Insight (2019) 4(24):e132975. doi: 10.1172/jci.insight.132975 PMC6975264 31751318

[108] Lewis ND Asim M Barry DP de Sablet T Singh K Piazuelo MB. Immune Evasion by Helicobacter Pylori Is Mediated by Induction of Macrophage Arginase II. J Immunol (2011) 186:3632–41. doi: 10.4049/jimmunol.1003431 PMC3069806 21296975

[109] McGovern N Shin A Low G Low D Duan K Yao LJ. Ginhoux, Human Fetal Dendritic Cells Promote Prenatal T-Cell Immune Suppression Through Arginase-2. Nature (2017) 546:662–6. doi: 10.1038/nature22795 PMC6588541 28614294

[110] Panfili E Mondanelli G Orabona C Bianchi R Gargaro M Fallarino F. IL-35Ig-Expressing Dendritic Cells Induce Tolerance *via* Arginase 1. J Cell Mol Med (2019) 23:3757–61. doi: 10.1111/jcmm.14215 PMC6484402 30793469

[111] Saini SK Hersby DS Tamhane T Povlsen HR Amaya Hernandez SP Nielsen M. SARS-CoV-2 Genome-Wide T Cell Epitope Mapping Reveals Immunodominance and Substantial CD8(+) T Cell Activation in COVID-19 Patients. Sci Immunol (2021) 6(58):eabf7550. doi: 10.1126/sciimmunol.abf7550 33853928 PMC8139428

[112] Hashimoto M Kamphorst AO Im SJ Kissick HT Pillai RN Ramalingam SS. CD8 T Cell Exhaustion in Chronic Infection and Cancer: Opportunities for Interventions. Annu Rev Med (2018) 69:301–18. doi: 10.1146/annurev-med-012017-043208 29414259

[113] Rha MS Jeong HW Ko JH Choi SJ Seo IH Lee JS. PD-1-Expressing SARS-CoV-2-Specific CD8(+) T Cells Are Not Exhausted, But Functional in Patients With COVID-19. Immunity (2021) 54:44–52.e3. doi: 10.1016/j.immuni.2020.12.002 33338412 PMC7834198

[114] Neefjes J Jongsma ML Paul P Bakke O. Towards a Systems Understanding of MHC Class I and MHC Class II Antigen Presentation. Nat Rev Immunol (2011) 11:823–36. doi: 10.1038/nri3084 22076556

[115] Roche PA Furuta K. The Ins and Outs of MHC Class II-Mediated Antigen Processing and Presentation. Nat Rev Immunol (2015) 15:203–16. doi: 10.1038/nri3818 PMC6314495 25720354

[116] McCusker D Wilson M Trowsdale J. Organization of the Genes Encoding the Human Proteasome Activators PA28alpha and Beta. Immunogenetics (1999) 49:438–45. doi: 10.1007/s002510050517 10199920

[117] Jung M Shin MK Jung YK Yoo HS. Modulation of Macrophage Activities in Proliferation, Lysosome, and Phagosome by the Nonspecific Immunostimulator, Mica. PloS One (2015) 10:e0117838. doi: 10.1371/journal.pone.0117838 25668030 PMC4323240

[118] Jutras I Houde M Currier N Boulais J Duclos S LaBoissiere S. Modulation of the Phagosome Proteome by Interferon-Gamma. Mol Cell Proteomics (2008) 7:697–715. doi: 10.1074/mcp.M700267-MCP200 18156134

[119] Zhao C Zhao W. NLRP3 Inflammasome-A Key Player in Antiviral Responses. Front Immunol (2020) 11:211. doi: 10.3389/fimmu.2020.00211 32133002 PMC7040071

[120] Boro M Balaji KN. CXCL1 and CXCL2 Regulate NLRP3 Inflammasome Activation *via* G-Protein-Coupled Receptor Cxcr2. J Immunol (2017) 199:1660–71. doi: 10.4049/jimmunol.1700129 28739876

[121] Xie S Chen M Yan B He X Chen X Li D. Identification of a Role for the PI3K/AKT/mTOR Signaling Pathway in Innate Immune Cells. PloS One (2014) 9:e94496. doi: 10.1371/journal.pone.0094496 24718556 PMC3981814

