# Supplementary material for: Monocytic-Myeloid Derived Suppressor Cells Suppress T-Cell Responses in Recovered SARS CoV2-Infected Individuals
Source: Front Immunol. 2022 Jun 24;13:894543. doi: 10.3389/fimmu.2022.894543 (PMC9263272; doi:10.3389/fimmu.2022.894543)
Supplement: Supplementary file 1 [file DataSheet_1.doc]

Monocytic-Myeloid Derived Suppressor Cells Modulate T-Cell Responses in Recovered SARS CoV2-Infected Individuals

Nadejda Beliakova-Bethell ^1,2^, Kathirvel Maruthai^3^, Ruijie Xu^4,5^ and Liliana C. M. Salvador^3,4,5^ and Ankita Garg^3*^

^1^ Department of Medicine, University of California San Diego, CA, USA

^2^ VA San Diego Healthcare System and Veterans Medical Research Foundation, San Diego, CA

^3^ Department of Infectious Diseases, College of Veterinary Medicine, University of Georgia, Athens, GA, USA

^4^ Institute of Bioinformatics, University of Georgia, Athens, GA, USA

^5^ Center for the Ecology of Infectious Diseases, University of Georgia, Athens, GA, USA

*Address correspondence to: Ankita Garg, PhD,

Department of Infectious Diseases

College of Veterinary Medicine

University of Georgia,

Athens, GA, 30606

USA

**Phone**: 706-542-4541

**Fax**: 706-542-5771

**E-mail**: [agarg@uga.edu](mailto:agarg@uga.edu)


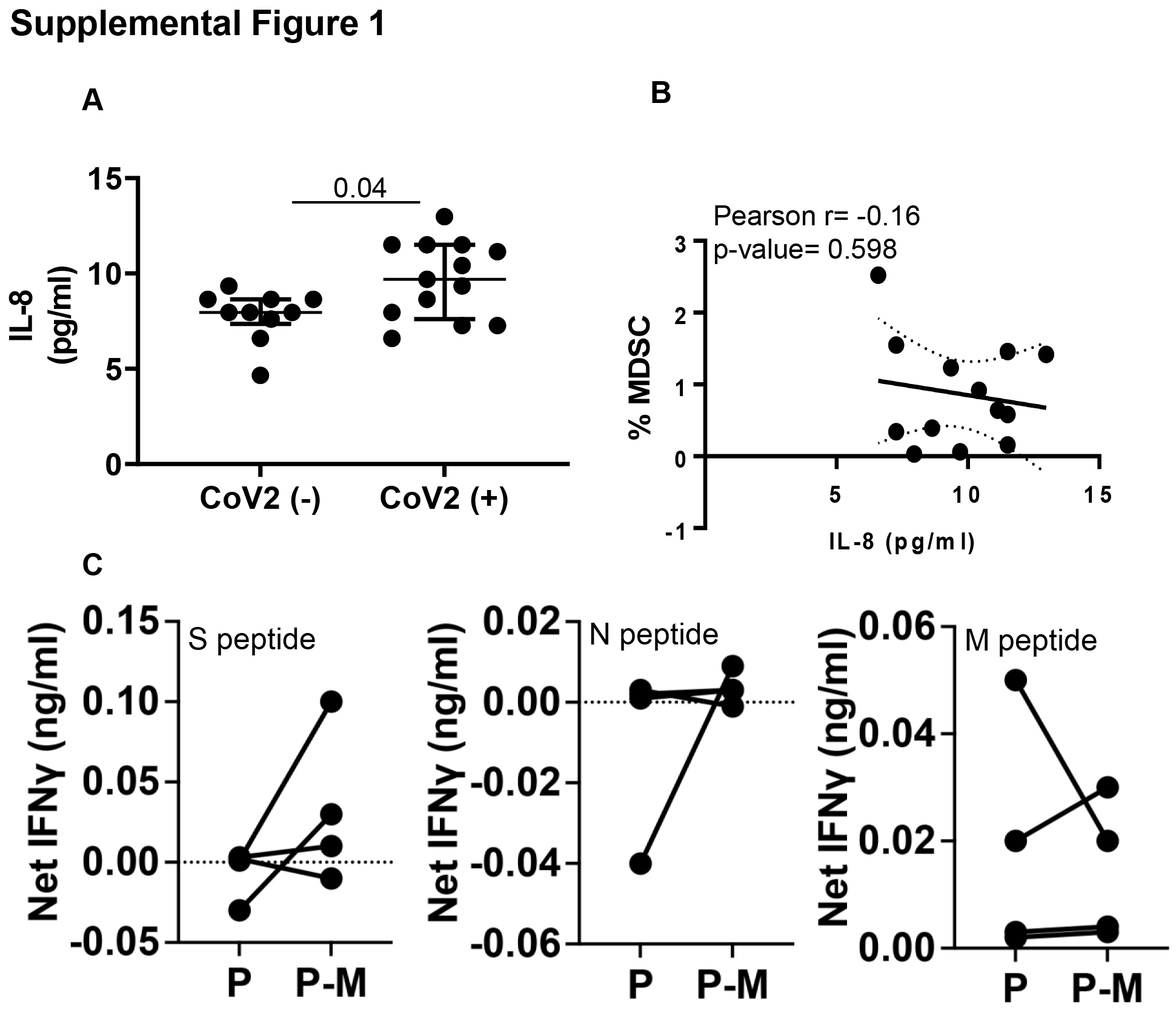


**(A-B) Plasma IL-8 is increased in CoV2+ individuals but does not correlate to M-MDSC frequency: (A)** The quantity of cytokine IL-8 in the plasma of CoV2- and CoV2+ individuals was measured by ELISA, as in Methods. Each dot in the plots depicts data of each individual donor, the plots include observations from 25^th^ to 75^th^ percentile. The horizontal line represents the median value. **(B)** Plasma IL-8 quantity was correlated with the circulating frequency of M-MDSC in CoV2+ individuals. Each dot in the plot depicts data of each individual donor; black solid and dotted lines, model-estimated values, and their 95% confidence intervals.

**(C) IFNγ production by CoV2- individuals in response to CoV2 peptide pools:** Freshly isolated PBMC from CoV2- individuals were stained with anti-CD14 and -HLA DR antibodies; CD14^+^HLA DR^-/lo^ M-MDSC were depleted from PBMC by flow cytometry. Whole PBMC (P) and MDSC depleted PBMC (P-M) were cultured in the absence or presence of peptide pools of S-, N-, and M- antigens of CoV2 for 48-72 hours. Culture supernatant was stored at -80^0^C until further use. The quantity of IFNγ in the culture supernatant was measured by ELISA. Each dot represents an individual donor

**Supplemental Figure 2**

**
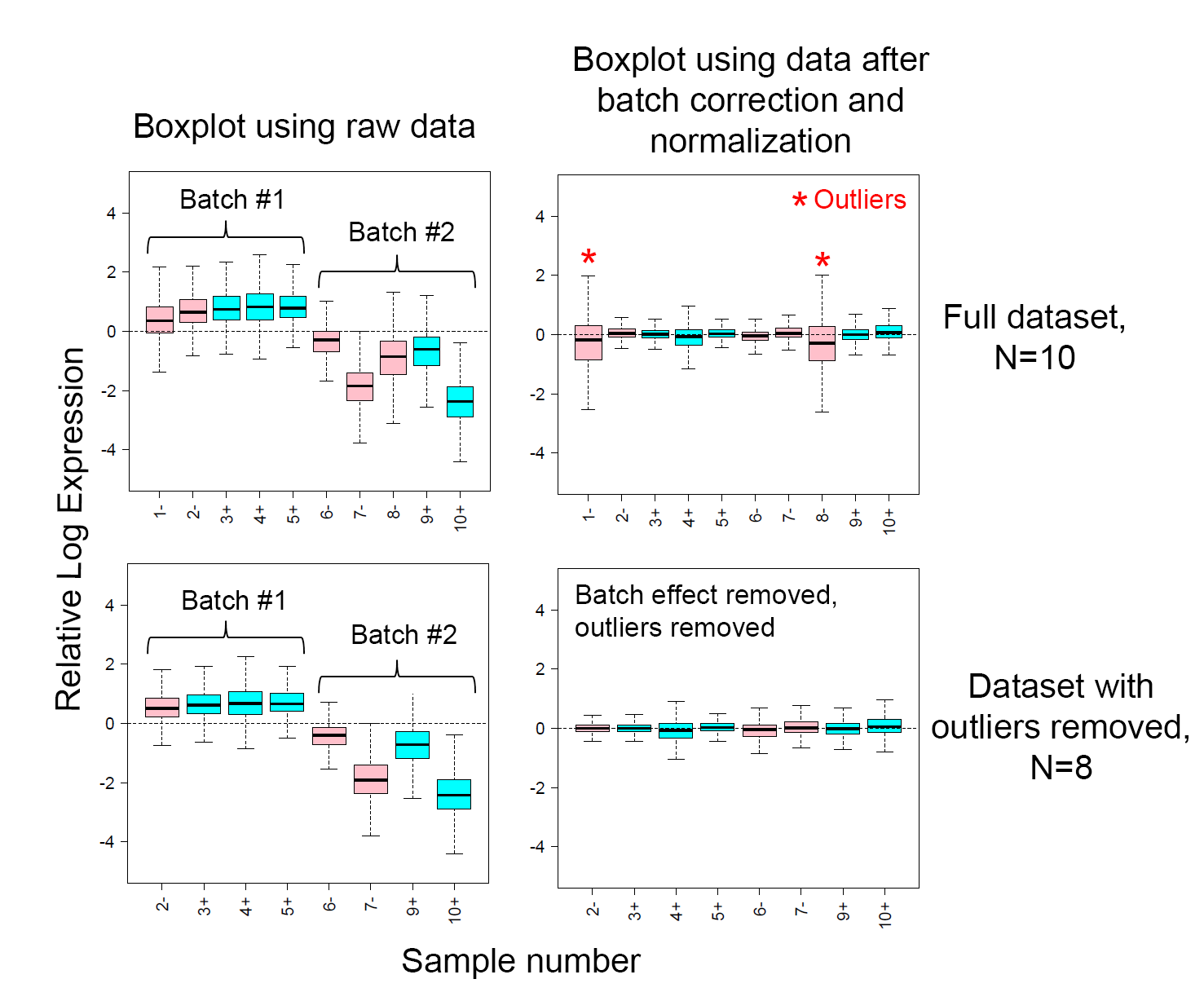
**

[

**The Relative Log Expression plot was used to assess for batch effects and outliers in the data.** This plot is a diagnostic tool to visualize the differences in the distribution of read counts across samples. Boxplots of the log-ratios of the gene-level read counts of each sample to those of the reference (median) sample are shown. Ideally, the distributions should be centered around zero and be as tight as possible (library *EDASeq* documentation). With the dataset that included all 10 samples (top, left), differences in the read distribution based on batch effect were clearly visible. After batch effect correction using *ComBat_seq* function in the library *sva* and upperquartile normalization (top, right), all samples centered around zero; however, two of the samples had different read distribution (red asterisk). After removal of these two outliers, batch effect was still obvious in the raw data (bottom, left), but after batch correction and upperquartile normalization, all samples tightly centered around zero line. Therefore, this dataset was subsequently used for differential gene expression analysis.

**Supplemental Figure 3**

**
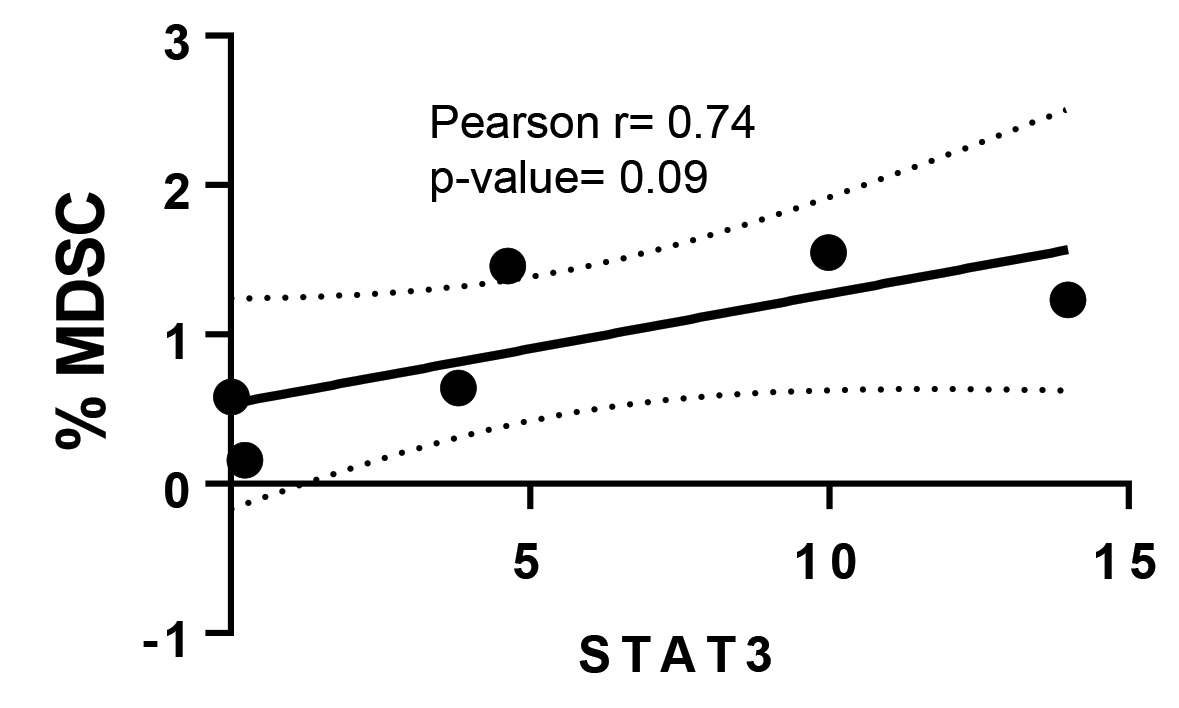
**

**M-MDSC correlate with STAT3 expression:** The circulating frequency of M-MDSC in peripheral blood (Figure 1B) was correlated with the gene expression of STAT3 in M-MDSC sorted by flow cytometry (Figure 6B) in a subset of CoV2+ individuals. ) Each dot in the plot depicts data of each individual donor; black solid and dotted lines, model-estimated values, and their 95% confidence intervals.

**Supplemental Figure 4**

**
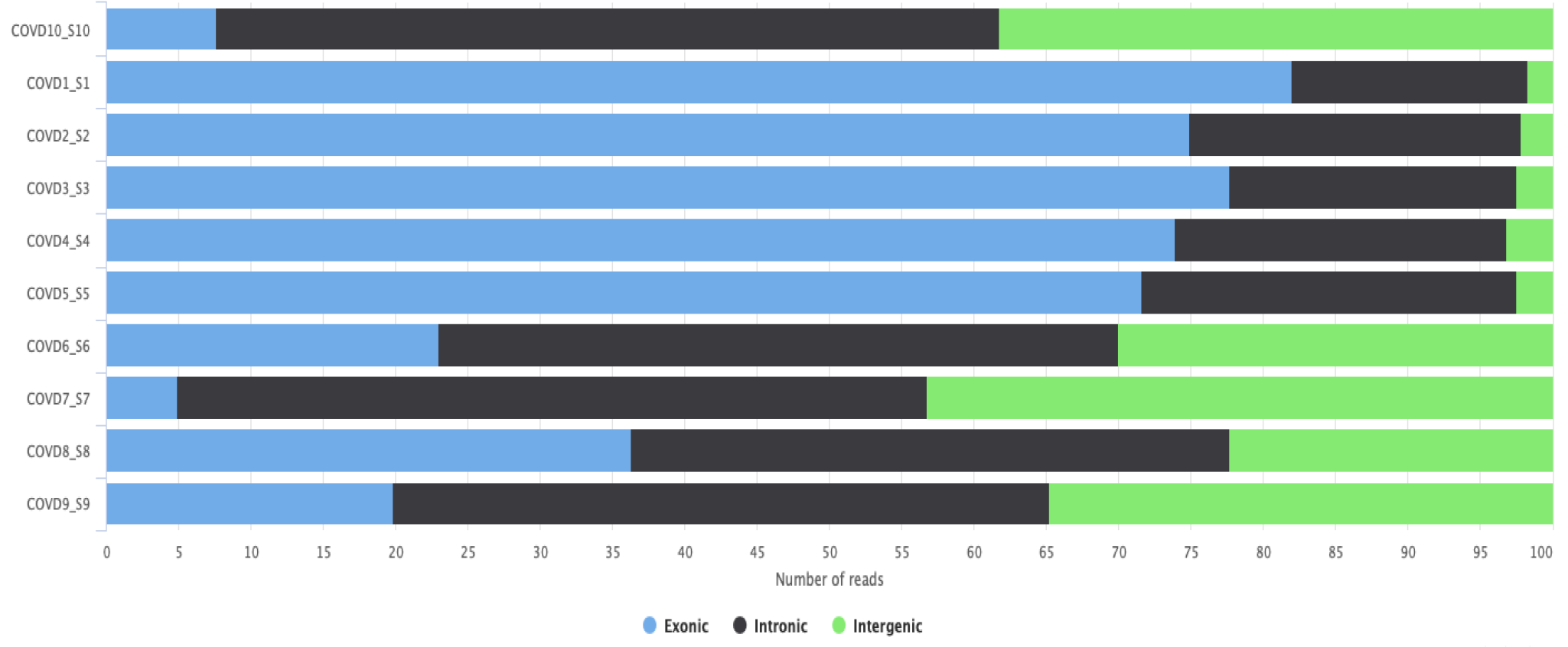
**

**Percentage of reads mapped to the exonic, intronic, and intergenic regions of the human genome**: RNA-seq reads were mapped to the reference human genome GRCh38 using STAR v. 2.7.3. The percentage of reads mapped to the exonic, intronic, and intergenic regions of genome was annotated using the Qualimap v.2.2.1 and visualized using MultiQC v.1.11. (+) sign behind sample names represent CoV2+ individuals, and (-) sign behind samples names represent CoV2- individuals. Sample CoV2_1 to CoV2_5 was sequenced in the first batch and sample CoV2_6 to CoV_10 was sequenced in the second batch. Reads in batch 2 samples has distinctively smaller percentage of reads mapped to the exonic regions of human genome than that of samples in the first batch.
