# Supplementary material for: Monocytic-Myeloid Derived Suppressor Cells Suppress T-Cell Responses in Recovered SARS CoV2-Infected Individuals
Source: Front Immunol. 2022 Jun 24;13:894543. doi: 10.3389/fimmu.2022.894543 (PMC9263272; doi:10.3389/fimmu.2022.894543)
Supplement: Supplementary Table 1 — Primers used in the study for targeted gene expression: M-MDSC were sorted by flow cytometry and stored in Trizol. RNA was isolated and gene expression of genes was determined by qRT PCR as detailed in Methods. The list provides the sequences and annealing temperature of the genes studied in the study. [file Table_1.doc]

**List of Primers used in the study**:

| ***Primer*** | **Sequence** | **Tm (^o^C)** |
| --- | --- | --- |
| *ARG1 F* | 5′-ACTTAAAGAACAAGAGTGTGATGTG-3′ | 56 |
| *ARG1 R* | 5′-GTCCACGTCTCTCAAGCCAA-3′ |
| *NOS2 F* | 5′-TCCCGAAGTTCTCAAGGCAC-3′ | 60 |
| *NOS2 R* | 5′-CATAGCGGATGAGCTGAGCA-3′ |
| *PD-1 F* | 5′-CTCAGGGTGACAGAGAGAAG-3′ | 58 |
| *PD-1 R* | 5′-GACACCAACCACCAGGGTTT-3′ |
| *P47^phox^ F* | 5′-AATGGCAGGACCTGTCGGAGAA-3’ | 60 |
| *P47^phox^ R* | 5′-CCTGTTCTCTGGATTGATCGCC-3’ |
| *CTLA-4 F* | 5′-AGACCTGAACACCGCTCCC-3′ | 60 |
| *CTLA-4 R* | 5′-GTCAGCCTGCCGAAGCACT-3′ |
| *PDL1 F* | 5′-TGCTGCCCTTCAGATCACAG-3′ | 56 |
| *PDL1 R* | 5′-GGGCATTGACTTTCAGCGTG-3’ |
| *IL-10 F* | 5′-TCAAGGCGCATGTGAACTCC-3′ | 56 |
| *IL-10 R* | 5′-GATGTCAAACTCACTCATGT-3′ |
| *ARG2 F* | 5′-CTGGCTTGATGAAAAGGCTCTCC-3′ | 60 |
| *ARG2 R* | 5′-TGAGCGTGGATTCACTATCAGGT-3′ |
| *STAT1 F* | 5′-ATGGCAGTCTGGCGGCTGAATT-3′ | 60 |
| *STAT1 R* | 5′-CCAAACCAGGCTGGCACAATTG-3′ |
| *STAT3 F* | 5′-CTTTGAGACCGAGGTGTATCACC-3′ | 60 |
| *STAT3 R* | 5′-GGTCAGCATGTTGTACCACAGG-3′ |
| *STAT5b F* | 5′-GCCACTGTTCTCTGGGACAATG-3′ | 60 |
| *STAT5b R* | 5′-ACACGAGGTTCTCCTTGGTCAG-3′ |
| *TGFb1 F* | 5′-TACCTGAACCCGTGTTGCTCTC-3′ | 60 |
| *TGFb1 R* | 5′-GTTGCTGAGGTATCGCCAGGAA-3′ |
